# Quantitative Prediction of Protein–Polyelectrolyte Binding Thermodynamics: Adsorption of Heparin-Analog Polysulfates to the SARS-CoV-2 Spike Protein RBD

**DOI:** 10.1021/jacsau.4c00886

**Published:** 2025-01-06

**Authors:** Lenard Neander, Cedric Hannemann, Roland R. Netz, Anil Kumar Sahoo

**Affiliations:** † Department of Physics, Freie Universität Berlin, Arnimallee 14, Berlin 14195, Germany; ‡ Institute of Chemistry and Biochemistry, Freie Universität Berlin, Takustraße 3, Berlin 14195, Germany

**Keywords:** proteins, polyelectrolytes, binding thermodynamics, friction, polymer elasticity, molecular simulation

## Abstract

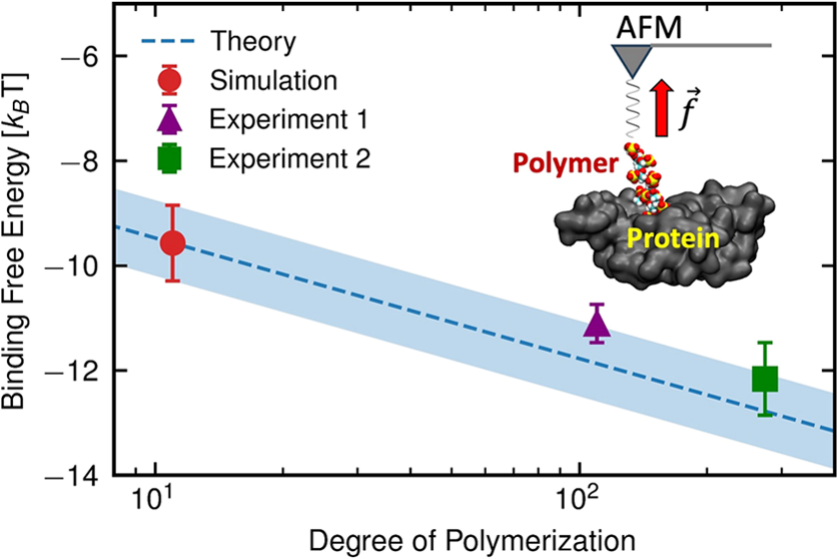

Interactions of polyelectrolytes (PEs) with proteins play a crucial role in numerous biological processes, such as the internalization of virus particles into host cells. Although docking, machine learning methods, and molecular dynamics (MD) simulations are utilized to estimate binding poses and binding free energies of small-molecule drugs to proteins, quantitative prediction of the binding thermodynamics of PE-based drugs presents a significant obstacle in computer-aided drug design. This is due to the sluggish dynamics of PEs caused by their size and strong charge–charge correlations. In this paper, we introduce advanced sampling methods based on a force-spectroscopy setup and theoretical modeling to overcome this barrier. We exemplify our method with explicit solvent all-atom MD simulations of the interactions between anionic PEs that show antiviral properties, namely heparin and linear polyglycerol sulfate (LPGS), and the SARS-CoV-2 spike protein receptor binding domain (RBD). Our prediction for the binding free-energy of LPGS to the wild-type RBD matches experimentally measured dissociation constants within thermal energy, *k*_B_*T*, and correctly reproduces the experimental PE-length dependence. We find that LPGS binds to the Delta-variant RBD with an additional free-energy gain of 2.4 *k*_B_*T*, compared to the wild-type RBD, due to the additional presence of two mutated cationic residues contributing to the electrostatic energy gain. We show that the LPGS–RBD binding is solvent dominated and enthalpy driven, though with a large entropy–enthalpy compensation. Our method is applicable to general polymer adsorption phenomena and predicts precise binding free energies and reconfigurational friction as needed for drug and drug-delivery design.

## Introduction

Understanding the interaction of polyelectrolytes (PEs) with proteins is important for interpreting structure formation in biology, e.g., in nucleosomes,^1−3^ intracellular condensates,^4^ and the brain,^5,6^ as well as elucidating different biological processes including viral infections or inhibitions.^7−9^ The cell entry of many viruses is mediated by the interaction of anionic heparan sulfate proteoglycans (HSPGs) present on the extracellular matrix of the host cell with positively charged viral surface glycoproteins.^10^ Due to this nonspecific electrostatic attraction, the concentration of virions at the cell surface is increased, making it more likely for them to bind to the host cell receptor proteins, which often includes multivalent interactions^11^ and eventually leads to viral invasion.^12^ This mechanism has been observed for different viruses such as Hepatitis B and C, herpes simplex virus, etc.^10^ and more recently for the SARS-CoV-2 virus, responsible for the COVID-19 pandemic.^13,14^ In the case of SARS-CoV-2, a cationic patch present on the spike protein receptor binding domain (RBD) binds to HSPGs,^15^ which makes it more feasible for the RBD to specifically interact with the host cell receptor, the angiotensin-converting enzyme 2 (ACE2).^16^

**Figure 1:**
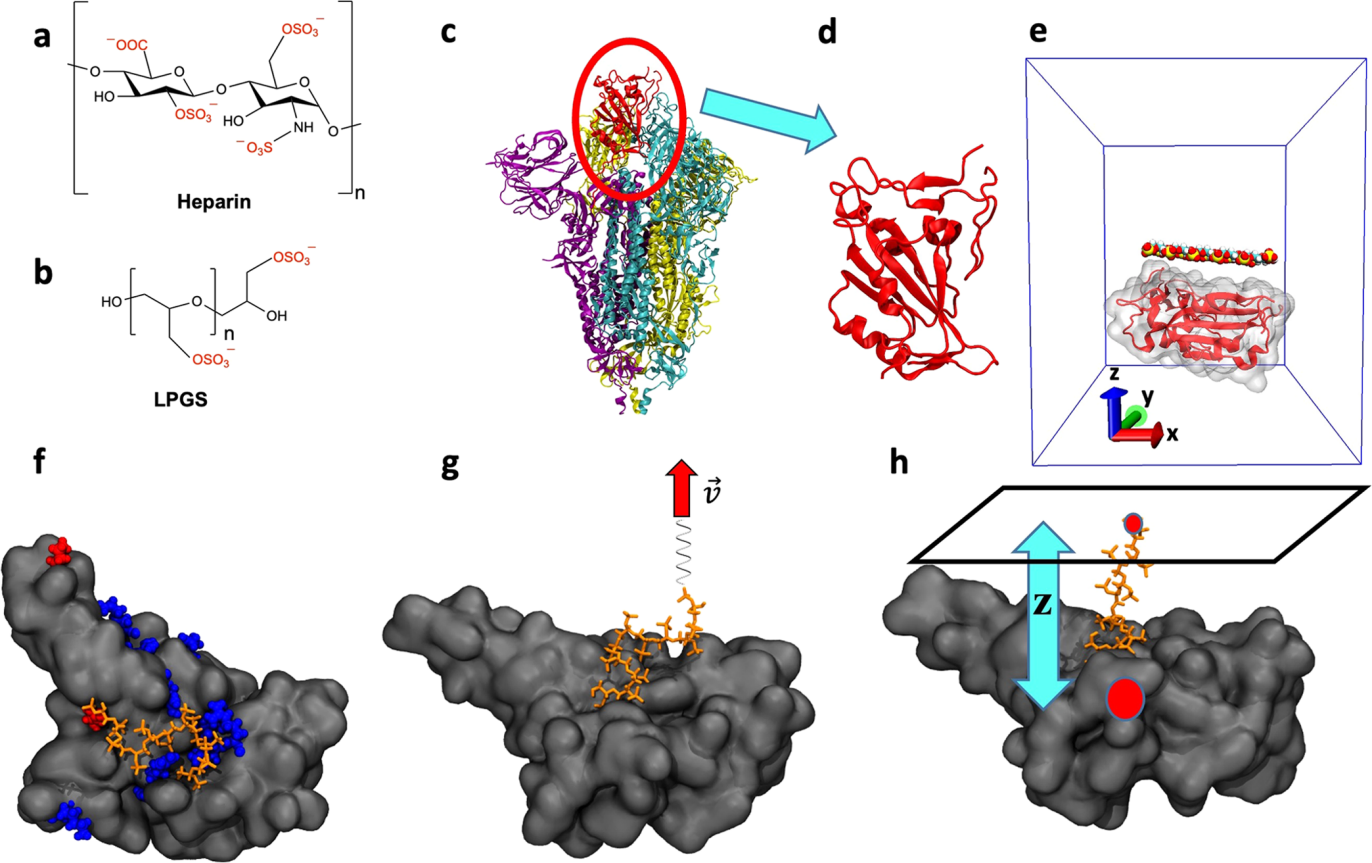
Simulation details. Chemical structures of (a) heparin and (b) linear polyglycerol sulfate (LPGS). (c) Structure of the spike protein trimer in the secondary structure representation (PDB ID: 7DK3).^32^ Monomers are shown in purple, cyan, and yellow. The receptor binding domain (RBD) of one monomer (cyan) is present in the up conformation and is shown in red. (d) Zoomed-in view of the RBD. (e) Simulation unit cell (blue box) for equilibrium adsorption of a polymer to the RBD (red). An LPGS undecamer is shown in the space-filling representation with color coding for the different atom types: hydrogen in white, carbon in cyan, oxygen in red, and sulfur in yellow. Water and ions are present but not shown for clarity. The *x*, *y*, and *z* axes are indicated with red, green, and blue arrows, respectively. (f) Simulation snapshot after 1000 ns of equilibration representing adsorption of the LPGS (shown in orange) to the Delta-variant RBD surface (shown in gray). Protein cationic residues are pointed out in blue, whereas mutated residues R452 and L478 are in red. (g) Dynamic pulling simulation protocol in which a spring connected to one of the terminal atoms of the polymer is pulled away from the protein surface with a constant velocity ν along the *z*-axis. The spring is free to move along the lateral direction. (h) Static pulling simulation protocol in which one of the terminal atoms of the polymer is allowed to freely move in a plane at constant z-separation (distance projected along the normal to the plane) from the protein center-of-mass (red circle).

The study of PE–protein interaction is also of major importance for the development of new drugs. Heparin, a naturally occurring anionic PE for which the chemical structure is shown in Figure 1a has been the subject of intense research in the last few decades due to its ability to adsorb at positively charged surfaces and thereby modulate biological processes.^17−19^

More recently it has been used to treat patients with SARS CoV-2 infections.^15,16,20,21^ By binding to the cationic patch of the RBD, heparin can compete with HSPGs and block the first step of the cell-entry process. However, heparin-based drugs come with anticoagulatory side effects, which can be a disadvantage for the treatment of specific diseases.^20,22^ Therefore, there is great interest in developing heparin analogs that share the same characteristic for adsorbing to cationic surfaces but with fewer side effects. Linear polyglycerol sulfate (LPGS), the chemical structure of which is shown in Figure 1b, has recently been tested to show excellent inhibitory activity against SARS-CoV-2, with significantly reduced cytotoxicity.^15^ LPGS, compared to heparin, shows larger binding affinities to the RBD of wild-type SARS-CoV-2 and its different variants,^15,23^ though having a lower linear charge density than heparin, details are provided in Figure S4 in the Supporting Information.

Computational methods developed in the last few decades have been quite successful in predicting binding poses and binding free-energies of small-molecule drugs with proteins.^24−28^ However, quantitative prediction of polymer–protein binding thermodynamics remains challenging because of the limited length scales and time scales accessible by atomistic simulations using present-day computational power.^29,30^ Specifically, PE-based viral inhibitors tested in experiments typically are 100–1000mers long with molecular weights of 10–100 kDa^15,31^ and the binding–unbinding equilibrium relaxation time for interactions between a charged monomeric unit and an oppositely charged protein residue can be a few microseconds.

In this article, we combine advanced sampling techniques and theoretical modeling to investigate the interaction of LPGS and heparin with the SARS-CoV-2 spike protein RBD using explicit solvent all-atom molecular dynamics (MD) simulations. Adapting a simulation setup that mimics atomic force microscopy experiments,^33,34^ we determine the PE–protein adsorption free energy from measuring the force to pull one terminus of the adsorbed PE away from the protein surface. By pulling this way, the intermolecular contacts are broken sequentially, overcoming a small free-energy barrier for each breakage, which leads to rather fast equilibration. The adsorption free energy thus obtained is compared with that computed using umbrella sampling simulations for validation. To obtain the standard binding free-energy of PEs, we add two correction terms: the PE stretching free energy due to the applied force and an entropic term accounting for its binding volume. For comparing the simulation results with experiments where longer PEs have been considered, we add the polymer translational entropy contribution in the bound state, which scales logarithmically in the degree of polymerization *N*, and find good agreement for the interaction of LPGS with the wild-type RBD compared with recent experimental measurements.^15,35^ Moreover, we decompose the free energy into enthalpic and entropic contributions arising from solute–solute and solute–solvent interactions and determine the PE–protein relative friction in the bound state, for a deeper understanding of the PE–protein binding thermodynamics and relaxation dynamics.

## Results and Discussion

Due to the large size of the trimeric SARS-CoV-2 spike protein, we consider in simulations only one monomer’s RBD (Figure 1c,d). The RBD binds to not only the HSPGs on the cell surfaces but also to the cell receptor protein ACE2. Thus, it plays a central role in the cell entry process of the virion and constitutes a suitable target for new drugs. Moreover, dissociation constants for PEs binding to a monomeric RBD have been reported from microscale thermophoresis experiments,^15,35^ making a meaningful comparison with the simulations possible. It should be noted that in vivo, there are glycans attached to the RBD,^36,37^ but far away from the cationic patch (the putative sulfate binding sites on the RBD),^15,23^ and hence these are not expected to affect the binding of anionic PEs to the RBD. We test this in a simulation (see Methods section for details) by attaching a glycan on top of the cationic patch and find LPGS to readily bind to the RBD, as shown in Figure S5 in the Supporting Information. This demonstrates that the entropic cost of replacing surface glycans is small compared to other interactions. Thus, we have excluded any glycan in our simulations for the results presented in the main text.

We perform simulations of LPGS interacting with both the wild-type RBD and the Delta-variant RBD with L452R and T478 K mutations. We take an LPGS undecamer in the simulations, much shorter than that used in experiments, to ensure fast equilibration. Additionally, we conduct simulations of a heparin pentamer interaction with the wild-type RBD, for which we observe a very long equilibration time, as will be discussed in Figure 6b,d. Therefore, we primarily focus in this study on simulation results involving LPGS binding and their quantitative comparison with experiments.

We start with unrestrained simulations (the setup shown in Figure 1e), where a polymer can move freely in the simulation box and interact with the protein surface. We observe that LPGS binds to the cationic patch of both the wild-type and Delta-variant RBD (shown in Figure 1f in blue). To validate that the adsorption to the cationic patch corresponds to the optimal binding position and is not an artifact of the starting position, we conduct six independent simulations, where we place LPGS at different starting positions as shown in Figure S6a in the Supporting Information. We find that within 400 ns LPGS binds to the same cationic patch in all six simulations, as shown in Figures S6b and S7 in the Supporting Information. Moreover, we calculate pairwise root-mean-square deviations (see eq 10 for the definition) of the RBD-bound LPGS conformations sampled in the different simulations and find significant overlaps among the corresponding distributions as shown in Figure S8. Therefore, the observed binding conformations are expected to be the minimum free-energy configurations and are suitable as starting structures for subsequent pulling simulations.

### Polymer Desorption Free Energy

**Figure 2:**
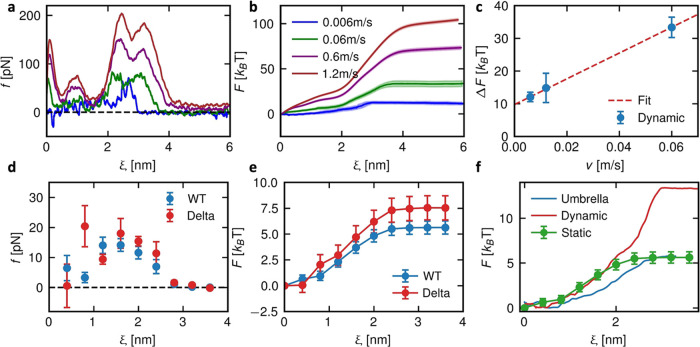
Simulation results for the desorption of an LPGS undecamer from the wild-type (WT) and, only if mentioned, Delta-variant RBD surface. (a) Force *f* and (b) free energy *F* profiles from dynamic pulling simulations, results for only four out of the six pulling velocities are displayed for visual clarity. Error bars are displayed as shaded colored areas in panel b. (c) Free-energy difference Δ*F* between the desorbed and adsorbed states as a function of the pulling velocity ν. The equilibrium free-energy difference Δ*F* (ν = 0) is obtained from linear extrapolation of the data for the three slowest pulling rates to zero velocity. (d) Force and (e) free-energy profiles from static pulling simulations. (f) Comparison of free energy profiles obtained from umbrella sampling and static pulling simulations with the dynamic pulling simulation result using the slowest pulling velocity ν = 0.006 m/s.

To obtain the LPGS desorption free-energy profile as a function of the reaction coordinate ξ, defined as the distance of the pulled polymer terminus from the protein surface (see Figure 1h), we use three different sampling techniques: dynamic pulling, static pulling, and umbrella sampling (see simulation details in Methods section). In the dynamic pulling simulations, a harmonic spring attached to one of the polymer’s terminal atoms is moved away from the protein surface along ξ at speed ν (see Figure 1g) and the force *f* acting on the spring is measured. Starting with the system configuration of LPGS adsorbed to the wild-type RBD, simulations are conducted with six different speeds ranging ν = 0.006 to 1.2 m/s. To make sampling comparable for different ν, we perform multiple simulations such that a total simulation time of at least 1 μs is reached for each ν. Mean desorption force profiles *f*(ξ) for different ν are shown in Figure 2a. We find that the most distinct force peaks are situated at ξ in the range of 2–4 nm and the desorption forces are higher for faster pulling speeds. The latter observation can be rationalized by friction effects originating from the force-induced breaking of hydrogen bonds and salt bridges.^38−40^ Free energy profiles *F*(ξ), obtained by integrating over the force profile as
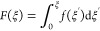
1are shown for different pulling speeds in Figure 2b. We find that apart from the higher free energy values for faster ν, the profiles for higher ν (= 1.2 and 0.6 m/s) do not reach plateau values, even after the complete desorption of the polymer. This shows that simulations are far from equilibrium for higher ν. As the friction contribution to the desorption force *f* in the viscous (i.e., low-velocity) regime is linear in *v*, the free-energy difference between the desorbed and adsorbed state, Δ*F* = *F*(ξ → ∞), increases linearly with ν according to Δ*F*(ν) = Δ*F*(ν = 0) + γν*L*_0_, where *L*_0_ = 4.03 nm (see Methods section) is the unstretched contour length of the polymer.^38^ We use this linear relationship to determine the equilibrium free energy of polymer desorption, Δ*F*(ν = 0), and the friction coefficient γ. Linear regression, using Δ*F* data for only the three lowest ν, leads to an excellent fit (Figure 2c), resulting in Δ*F*(ν = 0) = 9.7 ± 1.6 *k*_B_*T* and γ = 4.05 × 10^–10^ Ns/m (for results from fitting the whole data set, see Figure S9 in the Supporting Information). This is equivalent to a diffusion constant of *D* = *k*_B_*T*/γ = 10.23 nm^2^/μs, from which we obtain the diffusion time τ*_D_* = *L*_0_^2^/2*D* = 0.8 μs. We see that the bound polymer diffuses over its contour length in about a microsecond, allowing for rather quick equilibration of the bound state.

For the static pulling simulations, we select initial configurations from the dynamic pulling simulation at nine different ξ values with a spacing of 0.4 nm. At each ξ value, the polymer terminal atom is allowed only to freely move on a plane, maintaining a constant *z*-separation (distance projected along the normal to the plane) from the protein surface, see Figure 1h. The average force needed to keep the LPGS terminal atom at different ξ values is shown in Figure 2d for the wild-type and Delta-variant RBD. Compared to dynamic pulling results shown in Figure 2a, the peak force here is significantly lower. Also, the ξ value corresponding to the complete desorption of the polymer, represented by the force dropping to zero, is smaller than that observed in the dynamic pulling simulations. This is because the polymer is stretched more due to the larger pulling forces in the latter case. These deviations illustrate that even for the slowest pulling rate, the dynamic pulling simulation is far from equilibrium. Moreover, it is observed that forces for the Delta variant are significantly higher than the wild-type RBD at ξ = 0.8 nm. To understand this, we investigate the interaction of LPGS with individual protein residues as shown in Figure S10 in the Supporting Information, noting that conformational dynamics of wild-type and Delta-variant RBDs, whether free in solution or bound to LPGS, are very similar (see Figure S11 in the Supporting Information). For both RBD types, we find that LPGS forms a higher number of contacts with cationic residues such as R346 and R466. For the Delta variant, there is however an extra charged residue on the cationic patch (R452), which makes additional, electrostatically favorable contacts with LPGS simultaneously possible (Figure S10d in the Supporting Information), giving rise to the increased force. Free energy profiles show that the complete desorption of LPGS from the Delta-variant RBD, compared to the wild-type, requires an extra free energy of 2 *k*_B_*T* (Figure 2e). This demonstrates that cationic mutations in proteins lead to an increase in their binding affinities to anionic polymers, which supports the hypothesis of Nie et al.^23^ that the increased infectivities of Delta and Omicron variants are caused by additional cationic residues of the RBD that interact strongly with cellular HSPGs.

We also calculate the free energy profile of LPGS desorption from the wild-type RBD using umbrella sampling simulations with the weighted histogram analysis method (for details, see Methods section).^41,42^ Free energy profiles *F*(ξ) obtained from all three methods are compared in Figure 2f. *F*(ξ) from the dynamic pulling simulation using even the slowest pulling velocity, ν = 0.006 m/s, deviates significantly from the other two methods, while the extrapolated free-energy difference Δ*F*(ν = 0) to zero velocity exhibits less deviation from the umbrella sampling result, Δ*F* = 5.8 ± 0.5 *k*_B_*T*, and the static pulling result, Δ*F* = 5.6 ± 0.6 *k*_B_*T*.

### Dissociation Constant and Standard Binding Free Energy

The dissociation constant *K*_D_ for a complexation reaction between protein *P* and polymeric ligand *L*,
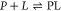
determines the ratio of concentrations of free, [*P*], [*L*], and bound, [PL], species in solution and is related to the free-energy change upon binding, Δ*F*_b_, and the ligand-binding volume *V*_b_ according to^43^
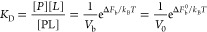
2Here, *V*_b_ represents the limited volume available for the polymer to move in the protein-bound state (for the procedure to obtain *V*_b_ from simulations, see the Supporting Information text and Figures S2 and S3). *V*_0_ = 1.661 nm^3^ is the standard-state volume corresponding to the concentration of 1 M and the standard free-energy of binding is given by Δ*F*_b_^0^ = Δ*F*_b_ – *k*_B_*T* ln (*V*_b_/ *V*_0_). Δ*F*_b_ is obtained from the polymer desorption free-energy Δ*F* by removing the polymer stretching free-energy due to the applied force, resulting in
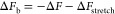
3

**Figure 3:**
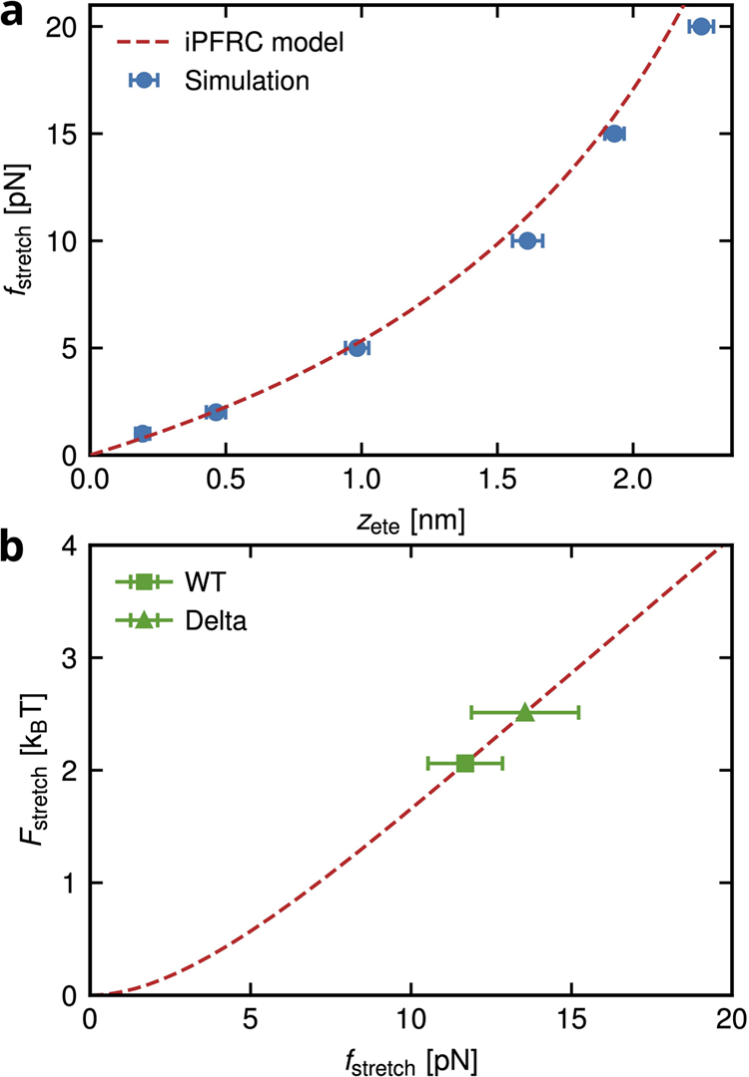
(a) Stretching force *f*_stretch_ versus the average end-to-end distance *z*_ete_ of an LPGS undecamer along the direction of applied force. Data points are fitted to the iPFRC model force–extension relation eq 8. (b) Stretching free-energy profile *F*_stretch_ as a function of *f*_stretch_, obtained by integrating the fitted curve in panel a.

Δ*F*_stretch_, in eq 3, describes the change in free energy to stretch the polymer from its relaxed state and is not present in the experiments and hence its contribution is subtracted from the simulated free energy. Δ*F*_stretch_ is obtained from constant-force stretching simulations of the polymer in free solution (details given in Methods section), by measuring the average end-to-end distances, *z*_ete_, projected along the direction of external force at different stretching forces, *f*_stretch_, applied to each end of the polymer. We fit the force–extension relation of the inhomogeneous partially freely rotating chain (iPFRC) model^44^ (for details, see Methods section) to the simulation values and find an excellent fitting as shown in Figure 3a. By integrating the force–extension relation from the relaxed end-to-end distance *z*_ete_^0^ = 0 to a stretched end-to-end distance *z*_ete_^stretch^,
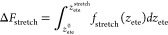
4we obtain the stretching free energy profile as a function of *f*_stretch_, shown in Figure 3b. To obtain Δ*F*_stretch_, we compute the average force experienced by a strained LPGS bound to the RBD (see the force plateau in Figure 2d from 1.2 to 2.4 nm) and take the stretching free energy at the average forces of *f*_stretch_ = 11.7 pN and 13.5 pN for the wild-type and Delta RBD, respectively (see Figure 3b, green points). Note that the resulting polymer stretching free-energy contribution, Δ*F*_stretch_ ≃ 2–2.5 *k*_B_*T*, is sizable.

Finally, the standard binding free-energy Δ*F*_b_^0^ as a function of the degree of polymerization, *N*, for longer polymers used in the experiments,^15,35^ compared to the simulations, *N* > *N*_sim_, can be extrapolated from simulations data as (for a derivation, see Section S1 in the Supporting Information)
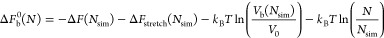
5Here, the last term represents the avidity entropy contribution, −*T*Δ*S*_avidity_, to the binding, as explained in the following.^31,45,46^ The direct polymer–protein interaction energy contribution to the binding free energy is limited to the number of binding sites *n*_b_ ≃ 5 on the protein, estimated to be the number of charged residues on the cationic patch of the RBD. Thus, LPGS longer than a critical length, equivalent to the size of the cationic patch, do not contribute to this direct interaction. There is, however, a combinatorial entropy contribution for a longer polymer with *N* > *n*_b_, because of the different ways the polymer can bind to the protein, given by *S*_avidity_ = *k*_B_ ln (N – *n*_*b*_ + 1). As this entropy contribution, *S*_avidity_^sim^ = *k*_*B*_ ln (*N*_sim_ – *n*_*b*_ + 1), is already accounted for in our simulations, we obtain in the limit of *N* > *N*_sim_ ≫ *n*_b_, Δ*S*_avidity_ = *k*_*B*_ ln (N/*N*_sim_).

For the binding of the simulated LPGS undecamer to the wild-type and Delta-variant RBD, values of Δ*F*_b_^0^ (*N*_sim_) = −Δ*F* – Δ*F*_stretch_ – *k*_B_*T* ln (*V*_b_/*V*_0_), noting that the avidity entropy term in eq 5 vanishes for *N* = *N*_sim_, and its different contributions are provided in Table 1 based on the static pulling results. The LPGS undecamer binds to the Delta RBD more strongly than the wild-type with an additional free-energy gain of around 2.4 *k*_B_*T*, as expected from the favorable electrostatic interaction discussed before.

**Table 1:** Values for the Standard Free-Energy of Binding Δ*F*_b_^0^ of an LPGS undecamer, *N*_sim_ = 11, to the Wild-Type and Delta-Variant RBD Based on the Static Pulling Simulation Results, Along with the Contributions in eq 5 to Calculate Δ*F*_b_^0^

contributions	WT	Delta
Δ*F*	5.59 ± 0.63 *k*_B_*T*	7.46 ± 1.17 *k*_B_*T*
Δ*F*_stretch_	2.06 ± 0.29 *k*_B_*T*	2.51 ± 0.41 *k*_B_*T*
*V*_*b*_	11.32 ± 2.02 nm^3^	11.89 ± 4.91 nm^3^
*k*_B_*T* ln (*V*_b_/ *V*_0_)	1.92 ± 0.11 *k*_B_*T*	1.97 ± 0.25 *k*_B_*T*
Δ*F*_b_^0^	–9.57 ± 0.72 *k*_B_*T*	–11.94 ± 1.30 *k*_B_*T*

**Figure 4:**
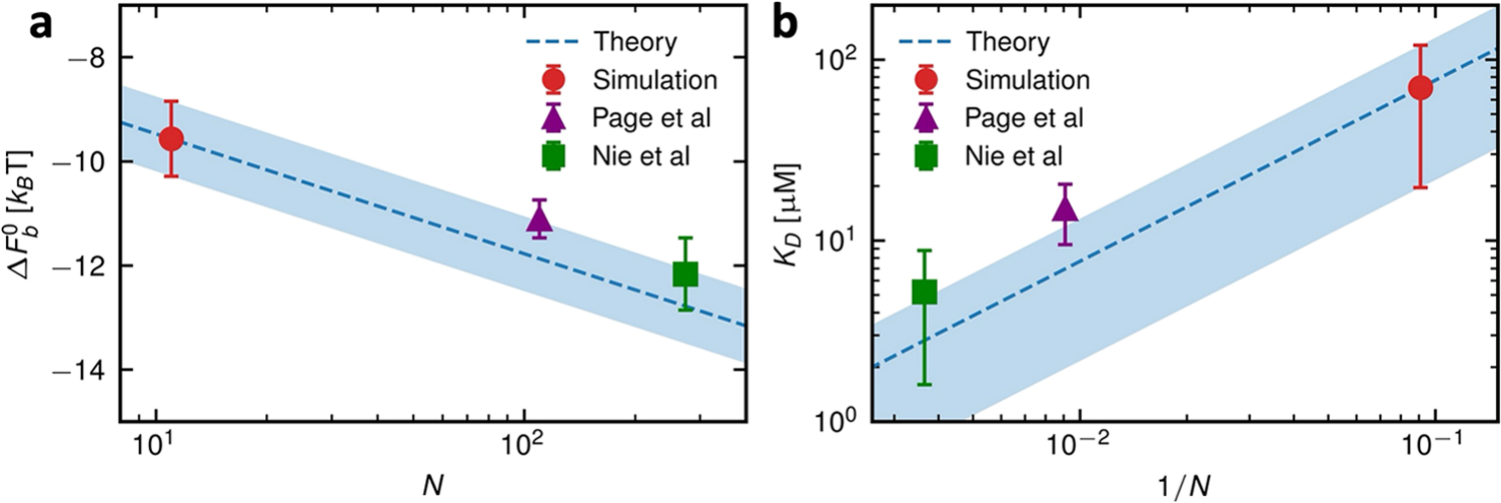
Theoretical prediction for (a) the standard binding free-energy Δ*F*_b_^0^ and (b) dissociation constant *K*_D_ for the complexation reaction of the wild-type RBD and LPGS as a function of its degree of polymerization *N*. Experimental values are reproduced from publications by Nie et al.^15^ (Available under a CC BY-NC 4.0 license. Copyright 2021 The Authors.) and Page et al.^35^ (Available under a CC BY-NC 4.0 license. Copyright 2023 The Authors.). Shaded regions in panels a and b represent simulation errors of propagation coming from the first three terms in eq 5 and from Δ*F*_b_^0^, respectively.

The standard binding free energy Δ*F*_b_^0^ and dissociation constant *K*_D_ for different polymer lengths predicted according to eqs 5 and 2 match nicely with the corresponding experimental values, as shown in Figure 4 and Table 2. The theoretical and experimental Δ*F*_b_^0^ values differ only within 1 *k*_B_*T* (the thermal energy), consequently *K*_D_ differ by a factor of roughly two.

**Table 2:** Comparison of Δ*F*_b_^0^ and *K*_D_ Values from Experiments and Theoretical Predictions for the Wild-Type RBD and LPGS Binding for Different Experimental Degrees of Polymerization *N*_exp_^a^

*N*_exp_	Δ*F*_b_^0^ (exp.)^b^	Δ*F*_b_^0^ (theory)^b^	*K*_D_ (exp.)^c^	*K*_D_ (theory)^c^
110 (Page et al.)	–11.11 ± 0.37	–11.87 ± 0.72	15.0 ± 5.5	7.0 ± 5.0
274 (Nie et al.)	–12.17 ± 0.69	–12.78 ± 0.72	5.2 ± 3.6	2.8 ± 2.0

a Experimental values are reproduced from publications by Nie et al., ref.^15^ (Available under a CC BY-NC 4.0 license. Copyright 2021 The Authors.) and Page et al., ref.^35^ (Available under a CC BY-NC 4.0 license. Copyright 2023 The Authors).

b Standard binding free-energy, Δ*F*_b_^0^ in *k*_B_*T*.

c Dissociation constant, *K*_D_ in μM.

### Enthalpy–Entropy Decomposition

**Figure 5:**
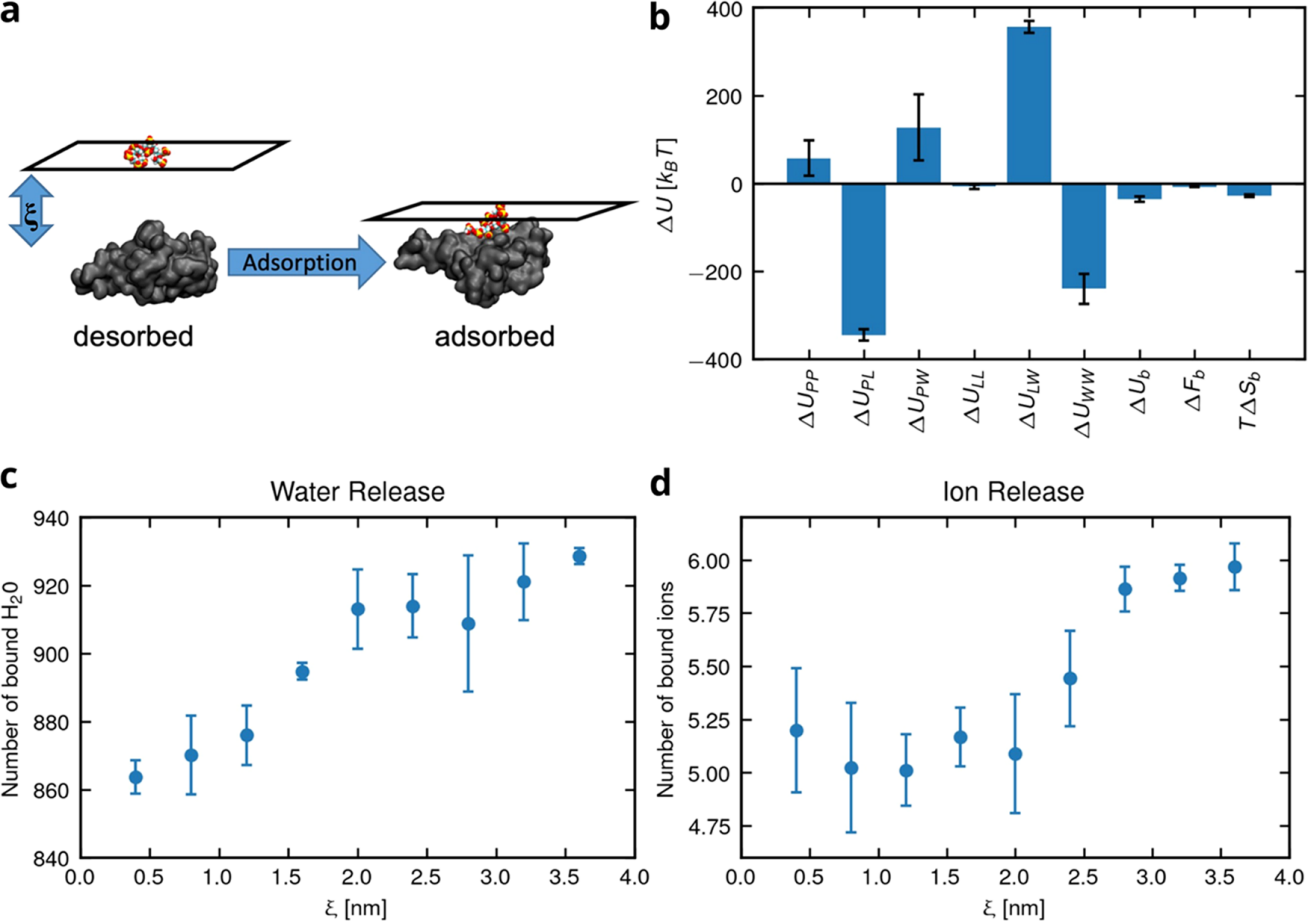
(a) Snapshots from the static pulling simulations for the desorbed state (at the pulling distance ξ = 3.6 nm) and the adsorbed state (ξ = 0 nm). (b) Enthalpic, Δ*U*_b_, and entropic, *T*Δ*S*_b_, contributions to the binding free-energy Δ*F*_b_ of the wild-type RBD–LPGS complex, along with the different internal energy contributions to Δ*U*_b_ (see text). The average number of (c) water molecules and (d) ions, Na^+^ and Cl^–^, bound to the RBD or LPGS as a function of ξ.

To understand the underlying contributions to the binding free energy Δ*F*_b_ = Δ*U*_b_ – *T*Δ*S*_b_, we decompose it into its enthalpic Δ*U*_b_ and entropic *T*Δ*S*_b_ parts, see Figure 5a,b. From the simulation trajectories (see Methods section), we calculate the net change in interaction energy, Δ*U*_b_ = Δ*U*_PP_ + Δ*U*_PL_ + Δ*U*_PW_ + Δ*U*_LL_ + Δ*U*_LW_ + Δ*U*_WW_, for the transition of the polymer from the desorbed to the adsorbed state from interactions among different components of the system: protein *P*, polymeric ligand *L*, and solvent (water molecules and ions) W. In the polymer adsorption process, we find favorable intersolute direct interaction (Δ*U*_PL_ < 0) and solvent reorganization energy (Δ*U*_WW_ < 0) and unfavorable solute–solvent interactions (Δ*U*_PW_ > 0 and Δ*U*_LW_ > 0). Δ*U*_b_ is quite small compared to the different contributions. Thus, there are huge cancellations among the different interaction energy contributions, as seen for other receptor–ligand systems,^33^ calling for highly accurate calculations of the solvent contribution.^47^

We obtain the entropy contribution to the binding using the thermodynamic relation Δ*S*_b_ = (Δ*U*_b_ – Δ*F*_b_)/*T*. By observing that Δ*U*_b_ < *T*Δ*S*_b_ < 0, we conclude that the adsorption process is entropically unfavorable and enthalpy driven. In the adsorption process, we find that 50–60 water molecules and only one ion are released as shown in Figure 5c,d (for the definition of bound water and ions, see Methods section). Thus, water release is expected to contribute significantly to the entropy as gain, while the contribution due to counterion release is minimal. The latter is not surprising though, as the linear charge density of LPGS is just above the counterion condensation limit by Manning,^48^ the length of the simulated LPGS is short, thus leading to end effects,^49^ and the simulated salt concentration of 150 mM is high.^50^ However, the net change in the binding entropy is negative (Figure 5b), which has to arise from the restricted conformational, translational, and rotational degrees of freedom of the polymer and protein in the bound state, overcompensating the entropy gain due to water and ion release.^50−53^ As the release of a single water molecule from a typical protein surface leads to an entropy gain of ∼1 *k*_B_*T*,^54^ the entropic loss due to conformational transformations of the protein and polymer is suggested to be greater than 60 *k*_B_*T*.

### Relaxation Time for Binding of Charged Groups

**Figure 6:**
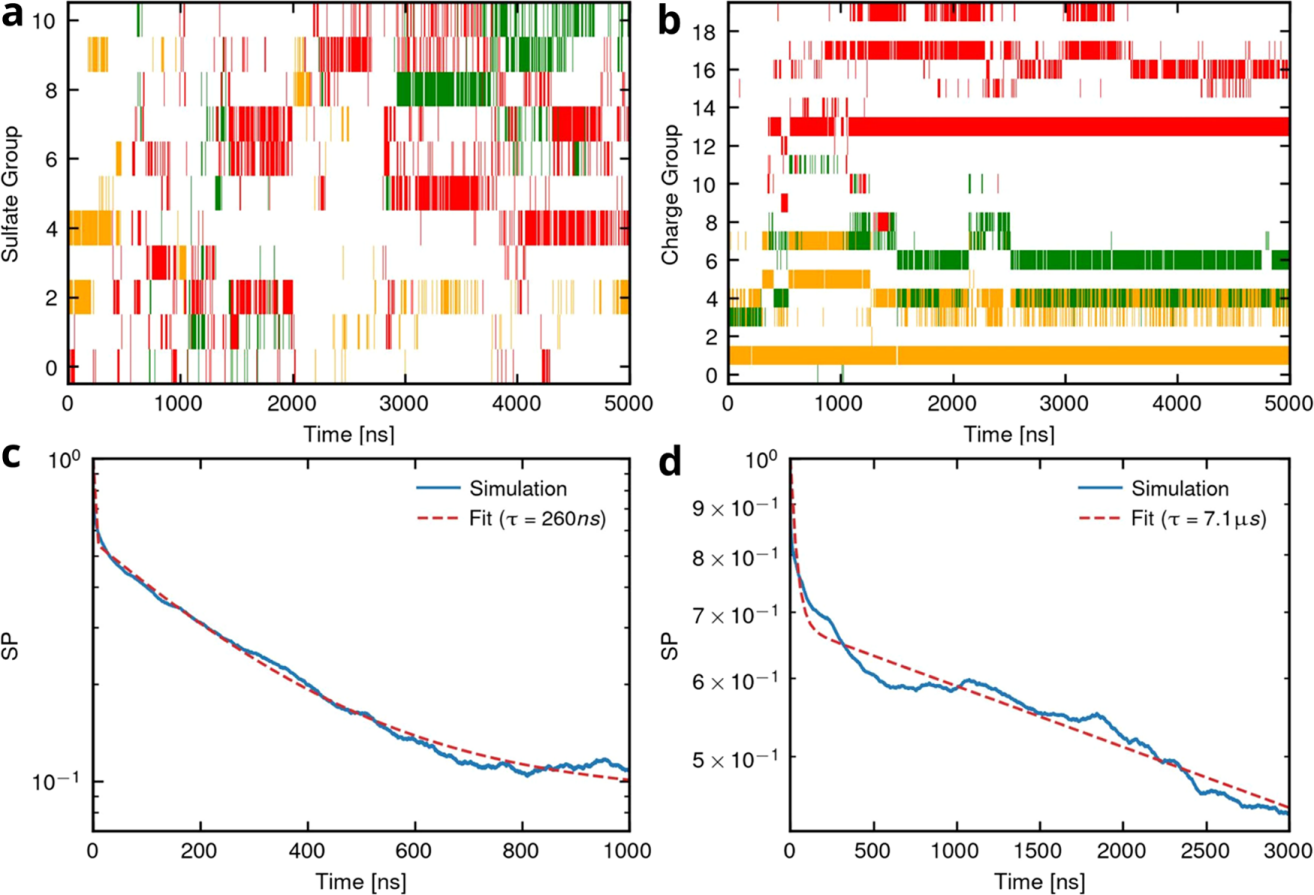
Time series for the binding of (a) LPGS’s and (b) heparin’s anionic groups (highlighted in red in Figure 1a,b) to cationic residues of the wild-type RBD that exhibit a significant number of close contacts with the polymers. Binding for LPGS (heparin) to the residues R346 (R346), K444 (R356) and R466 (R357) are visualized in red, green, and orange, respectively. The survival probability (defined in eq 6), averaging over all binding–unbinding time series data, is shown for (c) LPGS and (d) heparin. The dashed line in panel c or d represents the double exponential fit to the data, with the value of the largest decay time τ provided in the legend.

The validity of the binding free energy obtained from the static pulling simulations depends on whether the binding–unbinding equilibrium for interactions between the charged groups of the protein and polymer has been reached within the simulation time. The time-series data shows that anionic sulfate groups of LPGS bind intermittently to various cationic residues of the RBD (see Figure 6a). To quantify the time required to attain binding–unbinding equilibrium, we calculate the intermittent survival probability (SP) defined as^55^
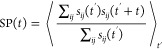
6where
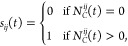
7with *N*_*C*_^*ij*^(*t*) being the number of close contacts (defined by an interatomic distance cutoff of 3.5 Å) between a polymer charge group *i* and a protein residue *j* at time *t*. SP(*t*) represents the probability of finding a polymer group that is bound to a protein residue at time *t*′ to be bound to the same residue at time *t*′ + *t* and is shown for LPGS interactions with the wild-type and Delta-variant RBD in Figures 6c and S12, respectively. The relaxation time τ refers to the largest time scale involved and is obtained by fitting a biexponential function (= *ae*^–*t*/τ_0_^ + *be*^–*t*/τ^ + *c*) to the SP data. τ values for LPGS bound to the wild-type and Delta-variant RBD are similar, around 250 ns, which is an order of magnitude smaller than the total simulation time of 5 μs at each ξ, ensuring sufficient sampling. Moreover, we check the convergence of the free-energy profile *F*(ξ) by splitting the simulation into five blocks, each of duration 1 μs, and calculating *F*(ξ) for each block, for LPGS binding to the wild-type or Delta-variant RBD (see Figure S13 in the Supporting Information).

For heparin, the time-series data in Figure 6b shows that charged groups of heparin stay bound to a single protein residue for almost the whole simulation time. From the SP function in Figure 6d we estimate a relaxation time of 7.1 μs, which suggests that an order of magnitude-longer simulation (∼50 μs) would be required to achieve heparin binding–unbinding equilibrium. The significantly slower relaxation dynamics of heparin bound to the RBD surface, despite having a lower binding affinity than LPGS in experiments, is reflected by the slower conformational dynamics of heparin free in solution (see Figure S14 in the Supporting Information). Besides, heparin is significantly stiffer than LPGS and has a persistence length of about 1.4 nm that is roughly three times longer than the one of LPGS, as we have shown in our recent paper.^15^ Thus, any reconfiguration of a protein-bound conformation of heparin involves large-scale rigid body rotations compared to the small-scale bending available to LPGS, which additionally is expected to slow down the relaxation of the RBD-bound heparin. Furthermore, to study the binding mechanism of heparin to the RBD we calculate contact maps of sulfate, carboxyl, and hydroxyl groups with the RBD residues. We find that heparin primarily interacts with the same set of cationic residues of the RBD, as the sulfates of LPGS, as shown in Figure S15a–d in the Supporting Information. Carboxyl groups form a greater number of close contacts with the RBD, primarily to its cationic residues, as shown in Figure S15e, whereas hydroxyl groups form hydrogen bonds with both cationic and polar residues, as shown in Figure S15f.

### Protein Conformational Transitions

**Figure 7:**
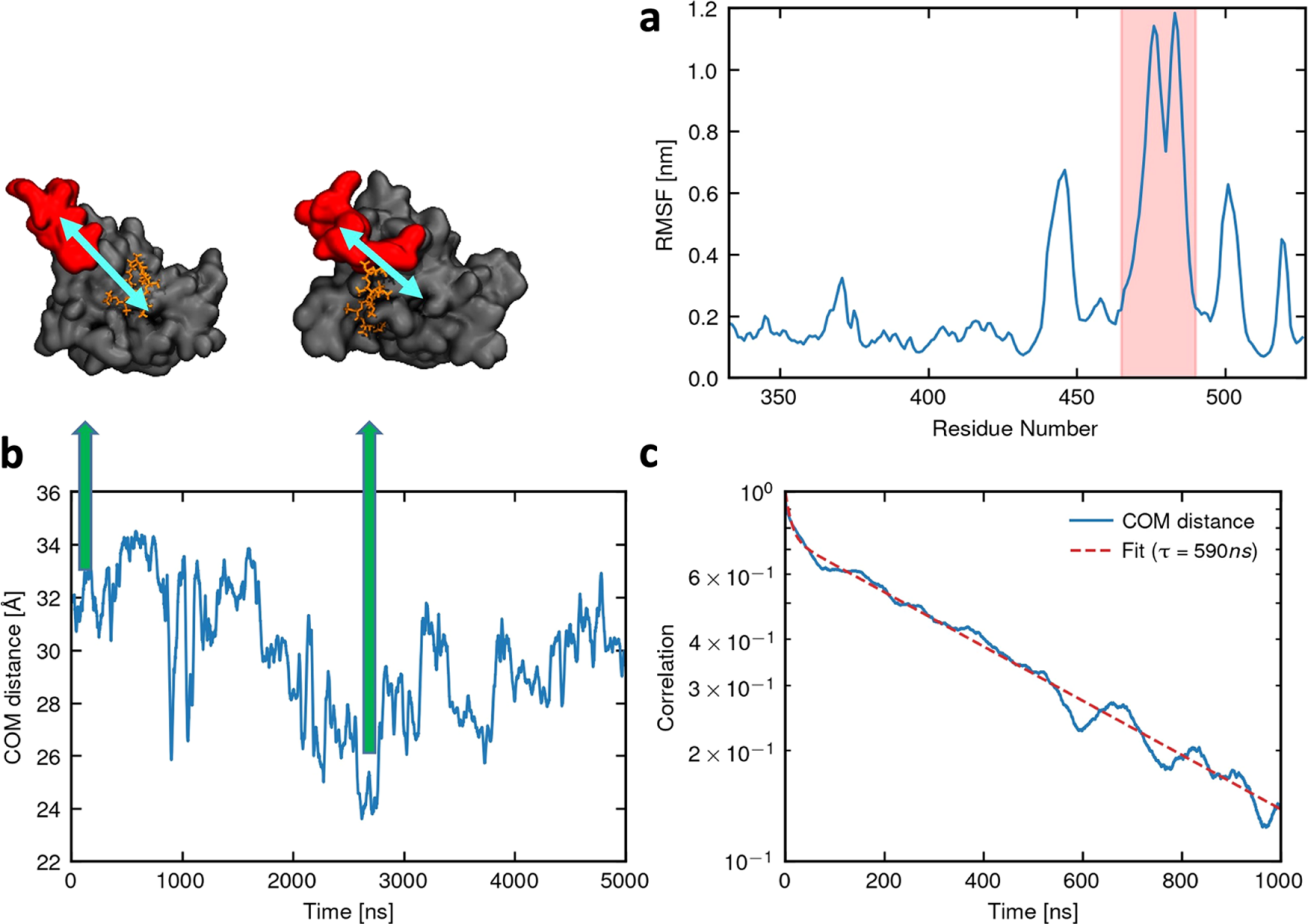
Conformational fluctuations of the wild-type RBD and dynamics of its loop region (residues 470 to 490) from the static simulation at ξ = 1.6 nm. (a) Root-mean-square fluctuation (RMSF) of the protein backbone atoms for different residues. The shaded region represents residues corresponding to the RBD’s loop region. (b) Time series of the distance between the center-of-mass of the loop region and the remaining part of the RBD. Snapshots for two different states at the start and after 2700 ns of the simulation are displayed above. The RBD surface is shown in gray except for its loop region in red, whereas LPGS is shown in the ball–stick representation in orange. (c) The center-of-mass distance autocorrelation function. The dashed line represents a double exponential fit to the data, with the value of the largest decay time τ given in the legend.

When LPGS is present near the wild-type RBD surface, we observe transitions between different protein conformations, which adds further complexity to the quantitative prediction of the binding thermodynamics. The RBD has a loop region (residues 470–490) that exhibits higher root-mean-square positional fluctuations (see eq 11 for the definition) and is highly flexible (see Figure 7a). Due to this, the loop region can switch between multiple states and thus modify the overall protein structure significantly, as seen from the time-series plot of the distance between the center-of-mass of the loop region and the remaining part of the protein in Figure 7b. From the autocorrelation function (defined in eq 12) of this center-of-mass distance, we find for the RBD conformational dynamics a relaxation time of 590 ns as shown in Figure 7c (for further details, see Methods section). As our simulation time is an order of magnitude larger than the relaxation time, sufficient sampling of different protein configurations is ensured. However, the error in the desorption force for the Delta-variant RBD is large (see Figure 2d, e.g., at ξ = 0.4 nm) since the bound polymer attracts the flexible loop of the protein toward it (see snapshots in Figure S16 in the Supporting Information).

Due to these protein structural transitions, also the error in the hydration number is large (Figure 5c, e.g., at ξ = 2.8 nm). Because the loop region occasionally adsorbs on the protein surface itself, multiple water molecules are released during this process (see Figure S17 in the Supporting Information). This accounts for large fluctuations in the number of bound water within a single simulation window, making a quantitative estimate of the entropic contribution due to water release difficult. Note that as the loop region is part of the receptor binding motif that forms direct contact with the host cell receptor protein ACE2, its flexibility might help in adapting the viral spike protein structure for binding to other cell receptors and thus improve viral infectivity.

## Conclusions

We demonstrate a method to obtain the binding free energy of long polymers (10–100 kDa) typically considered in experiments, from the simulated free-energy profile of shorter polymer desorption from a protein. This requires correctly accounting for (i) the binding volume of the polymer, (ii) the polymer stretching free energy, and (iii) the avidity entropy due to the different possible ways the polymer can bind to the protein (cf. eq 5). We validate our method by favorable comparison with the experimental free-energy of binding between LPGS and the wild-type SARS-CoV-2 spike protein RBD and reproducing accurately the polymer-length dependence. We find that anionic LPGS binds more strongly to the Delta-variant RBD (with extra 2 mutated cationic residues) than the wild-type RBD, underlining the role of electrostatic interactions. The LPGS–RBD binding at *T* = 300 K is found to be enthalpy driven with a large enthalpy–entropy compensation. Decomposing the enthalpy of binding, we find significant cancellation between the polymer–protein, the solute–solvent, and the solvent–solvent interaction energy contributions. These observations signify the importance of solvent and entropic effects in molecular binding.^33,54,56^

We identify a highly flexible loop region of the RBD, which transitions between different states with a relaxation time of 600 ns. Thus, slow protein conformation transitions can add complications in predicting the binding thermodynamics accurately. Moreover, we show that modeling the adsorption of a highly charged polymer, e.g., the drug heparin to RBD, requires a high computational effort as the relaxation time for the charged-group binding equilibrium is ∼7 μs.

Comparing three different simulation protocols (see additional discussion in Section S2 in the Supporting Information) for obtaining the polymer adsorption free-energy difference, we find that the extrapolation method using the dynamic pulling data, as shown in Figure 2, gives larger values than umbrella sampling and static pulling results. This hints at the relevance of dissipative chain reconfiguration effects when pulling a polymer from an absorbing surface, as is relevant in force spectroscopy experiments^57^ and biological nonequilibrium scenarios. Thus, the dynamic pulling method is not only useful in generating initial configurations for the static pulling method but also for understanding friction and diffusion in the bound protein–polymer complex, which is important for the kinetics of the binding process.

We have developed a theoretical method to predict the dissociation constant for a multivalent linear polymer binding to a monovalent receptor, i.e., a single RBD in solution, mimicking recent experiments where the binding of polymers to monomeric RBDs in solution was studied.^15,35^ A long polymer, however, can simultaneously bind to three RBDs (either in the up or down configuration) of a single trimeric spike protein or can bridge between multiple trivalent spike-protein receptors on the virion. Predicting multivalent dissociation constants in these two scenarios is possible with our recently developed multiscale modeling approach,^11,58^ for which the monovalent dissociation constant calculated in this paper is the essential input parameter.

## Methods

### MD Simulations

#### Models, Parameters, and Simulation Set-Up

The coordinates for the wild-type RBD of the SARS-CoV-2 spike protein are obtained from the deposited crystal structure (PDB ID: 6M0J).^21^ The Delta-variant RBD with L452R and T478 K mutations is built using PyMOL. The structure of the heparin pentamer is built using CHARMM-GUI Glycan Reader & Modeler.^59−62^ The structure of the LPGS undecamer is built using Avogadro software.^63^ CHARMM36m^64^ and CHARMM Carbohydrates^65,66^ force field parameters are used to model the protein and heparin, respectively. Parameters and partial atomic charges for LPGS are modeled with the CHARMM General force field^67,68^ and obtained using the CGenFF program.^69,70^ CHARMM-compatible TIP3P water^71,72^ and ion parameters^73^ are used. RBD/LPGS and RBD/heparin are arranged and solvated in boxes of sizes 7 × 7 × 9.5 and 7 × 7 × 10 nm^3^, respectively. Enough Na^+^ ions are added to charge neutralize each system, then Na^+^/Cl^–^ ion pairs are added to obtain a 150 mm NaCl solution estimated from the mole fraction of ion pairs and water.

Before starting pulling simulations, unconstrained simulations are performed for at least 1 μs in the NpT ensemble at *T* = 300 K and *p* = 1 bar with periodic boundary conditions in *xyz* directions, using the GROMACS 2020.6 package.^74^ During the simulations, backbone atoms of three residues of the RBD are fixed to stop its center-of-mass translation and its rotation around the principal axes. The stochastic velocity rescaling thermostat^75^ with a time constant of τ = 0.1 ps is used to control the temperature, while for the pressure control an isotropic Parrinello–Rahman barostat^76^ is used with a time constant of τ = 2 ps and a compressibility of κ = 4.5 × 10^–5^/bar. The LINCS algorithm^77^ is used to constrain the bonds involving H-atoms, allowing a time step of Δ*t* = 2 fs. Electrostatic interactions are computed using the particle mesh Ewald method^78^ with a real-space cutoff distance of 1.2 nm, while van der Waals interactions are modeled using Lennard-Jones potentials with a cutoff distance of 1.2 nm where the resulting forces smoothly switch to zero between of 1 and 1.2 nm. A data saving frequency of 100 ps is used.

#### Glycan-Conjugated RBD

A glycan (chemical details in Figure S5a in the Supporting Information) conjugated to the residue N354 of the wild-type RBD is built using the CHARMM-GUI Glycan Reader & Modeler.^60−62^ Parameters for the glycan are taken from the CHARMM Carbohydrates^65,66^ force field.

#### Dynamic and Static Pulling Simulations

For performing pulling simulations, the *z*-distance between the center-of-mass of the protein and one terminal atom of the polymer is chosen as a reaction coordinate ξ, which is shifted such that ξ = 0 when the terminal atom is bound to the surface. Constant velocity pulling simulations (which we refer to as dynamic pulling) are conducted in the NVT ensemble. Here, a spring with spring constant *k* = 1660 pN/nm is attached to a terminal polymer atom and pulled with a constant velocity v, and the pulling force *f* is obtained from the extension of the spring from its equilibrium position. The used pulling speed v ranges from 0.006 to 1.2 m/s. To ensure that the whole polymer is desorbed from the protein surface, a simulation time in the range of 5 to 1000 ns is used so that at the end, a pulling distance of roughly 6 nm is reached. For each pulling speed, simulations are repeated until the combined simulation time reaches 1000 ns or more. The coordinates of the system are saved every 100 ps and pulling forces are recorded every 100 fs.

For the static simulations, the starting configurations are generated from multiple configurations from the dynamic pulling simulations with a 0.4 nm spacing of the reaction coordinate. The time for each static simulation window is set to 5 μs, summing up to a total simulation time of 45 μs. The free-energy profile from the static simulations is calculated by first computing the average force for each simulation window using the last 4.5 μs data and then integrating over the average force profile, see eq 1.

#### Umbrella Sampling Simulations

Taking configurations from the dynamic pulling simulations for the wild-type RBD–LPGS system, we have conducted 30 simulations for 100 ns each where umbrella or harmonic potentials are applied to restrain the system at different values of the reaction coordinate ξ (the same as defined before), from 0.1 to 3.0 nm with a window spacing of 0.1 nm. The same spring constant as in the dynamic pulling is used for each umbrella window. The weighted histogram analysis method,^41^ implemented in the GROMACS module wham,^74^ is used to obtain the free-energy landscape *F*(ξ) for LPGS desorption from the RBD surface, shown in Figure 2f. The first 20 ns data for each umbrella window is discarded in the calculation of *F*(ξ). The overlapping of histograms for consecutive umbrella windows needed for the accurate computation of *F*(ξ) and its convergence check by taking different lengths of the simulation data are shown in Figure S18 in the Supporting Information. Note that the simulation time for each umbrella window is shorter than the RBD loop relaxation time reported in Figure 7c. However, the good comparison with the free-energy profile obtained from the static pulling simulations, each of a much longer duration of 5 μs, demonstrates that the umbrella simulations are converged (see Figure 2f).

#### Constant-Force Stretching of LPGS

An LPGS undecamer, placed in a rectangular simulation box of size 5 × 5 × 9 nm^3^, charge-neutralized by adding counterions (Na^+^) and solvated with water in a 150 mM NaCl solution, is taken for the simulations. The simulation-related parameters coincide with the ones of the unconstrained RBD/LPGS simulation in the NpT ensemble except, that the GROMACS 2021.3 package^74^ and ion parameters of Loche et al.^79^ are used. Additionally, we have chosen a higher trajectory saving frequency of 10 ps. The equilibration process starts with employing the steepest descent algorithm for the initial energy minimization. This is followed by two stages of simulation: a 500 ps NVT simulation and a 2 ns NpT simulation, during which the polymer atom positions are restrained.

**Figure 8:**
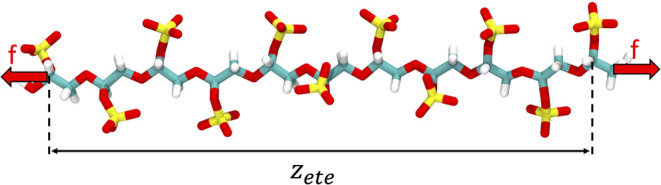
Stretching protocol showing the LPGS undecamer at an applied force *f* = 800 pN. Color coding for the different atom types: hydrogen in white, carbon in cyan, oxygen in red, and sulfur in yellow.

To determine the stretching free-energy Δ*F*_stretch_ of LPGS, we have performed in the NpT ensemble several production simulations in each of which a constant force between 1 and 1000 pN is applied to the polymer ends in opposite directions along the *z*-axis. The anchor points for the constant force are the first (C1) and last (C22) carbon atoms of the LPGS undecamer along its backbone, as depicted in Figure 8. The average extension ⟨*z*_ete_⟩ for ten monomers, defined as the distance between the carbon atoms C1 and C21 in the pulling direction, is measured. This ensures the inclusion of all relevant monomers under strain. We have performed NpT production simulations for different durations from 200 ns (at higher forces) to 2000 ns (at lower forces), depending on the relaxation time of the ion distribution around the polymer and the polymer end-to-end distance at different applied forces. For data analysis, the initial part (20–50 ns depending on the applied force) of a production run is discarded for the equilibration which accounts for initial stretching or shortening of LPGS.

### Simulation Data Analysis

Simulation data are visualized and analyzed with VMD^80^ and the software package MDAnalysis,^81,82^ respectively.

### iPFRC Model and Stretching Free Energy of LPGS

The force *f* versus extension *z*_ete_ profile obtained from constant-force stretching of LPGS is depicted in Figure 3a (for the complete range of forces used in this study, the profile is shown in Figure S19 in the Supporting Information). To interpolate the data points, we use the heuristic force–extension relation of the iPFRC model^44^
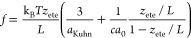
8Here, *L* is the contour length of the polymer, and *a*_0_ is the equilibrium monomer length. The Kuhn length *a*_Kuhn_ is defined by the linear stretching response at low applied forces and *c* is a free parameter, whose choice accounts for restricted backbone dihedral rotation and side chain interactions. The iPFRC model has been shown to describe the force–extension relation of various polypeptides quite well,^83^ when a force-dependent contour length *L*(*f*) is introduced additionally
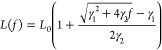
9In eq 9, *L*_0_ denotes the unstretched contour length of the polymer. The linear stretching modulus γ_1_ and the nonlinear coefficient γ_2_ describe the force-dependent extension of a monomer at zero temperature in vacuum. The variables *a*_0_, *a*_Kuhn_, *c*, γ_1_, and γ_2_ have been used as free fitting parameters and their values corresponding to the shown line in Figure 3a are reported in Table 3. The stretching free energy Δ*F*_stretch_ follows by integrating the fitted force–extension curve eq 4 and is shown in Figure 3b. The choice of setting the lower bound of the integral in eq 4 to zero is grounded on the premise that, in the absence of an applied force, the expected value of the average extension is zero.

**Table 3:** Fitting Parameters of the iPFRC Model

parameter	value
*a*_0_	366.82 ± 0.67 pm
*a*_Kuhn_	0.873 ± 0.033 nm
*c*	0.793 ± 0.018
γ_1_	96 ± 36 nN
γ_2_	500 ± 2000 nN

### Internal Energy Decomposition

Interaction energy calculations are done using the GROMACS module energy.^74^ The average energy is calculated for the adsorbed state, ξ = 0 nm, and the desorbed state, ξ = 3.6 nm, for interactions between different components of the system: protein, polymer, and solvent (water and ions), and the energy differences are computed. Only the short-range part of the Coulomb interaction with a cutoff distance 1.2 nm is included in these calculations, as the full, long-range electrostatic energy of a subsystem with a nonzero net charge with periodic boundary conditions diverges. However, calculation of the net change in interaction energy, Δ*U*_b_, for the whole system includes also the long-range electrostatic contribution. Simulation data of 2.5 μs (25,000 frames) and 5 μs (50,000 frames) are used for calculating the interaction energies for adsorbed and desorbed states, respectively.

### Distance Criteria for Close Contacts and Bound Water and Ions

For calculating the number of close contacts, we define a contact by an atom of LPGS falling within 3.5 Å of any atom of a protein residue. The same cutoff distance (of 3.5 Å) criterion is used to calculate the protein-bound water molecules and ions.

### Root-Mean-Square Deviation

The root-mean-square deviation (RMSD) of a molecular conformation is calculated using the GROMACS module *rmsd*(74) and is defined as
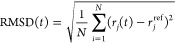
10where *N* is the total number of atoms, ***r***_*j*_(*t*) is the position of atom *j* at time *t*, and ***r***_*j*_^ref^ is the position the same atom in the reference state.

### Root-Mean-Square Fluctuation of the RBD Structure

The root-mean-square fluctuation (RMSF) of the backbone atom positions for each RBD residue is calculated using the GROMACS module *rmsf*(74) and is defined as
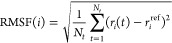
11where *N*_*t*_ is the total number of frames in a trajectory, ***r***_*i*_(*t*) is the position of residue *i* at time *t*, and ***r***_*i*_^ref^ is the position the same residue in the reference state (the native state of RBD).

### Relaxation Time for the RBD’s Loop Region Movement

The movement of the flexible loop region (residues 470–490) of the RBD is tracked by calculating the center-of-mass (COM) distance between the loop region and the rest of the protein. To get an estimate of the relaxation time, we calculate the COM distance autocorrelation function *C*(*t*) defined as
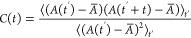
12where *A*(*t*′) is an observable at an initial time *t*′, *A* is the time average of observable *A*, ⟨·⟩_*t*′_ represents the time-origin averaging. The relaxation time is estimated by fitting a biexponential function (= *ae*^–*t*/τ_0_^ + *be*^–*t*/τ^) to the COM distance autocorrelation *C*(*t*) and refers to the longest time scale involved τ.

### Error Estimations

Errors for the static pulling, the average extension of LPGS, internal energy calculations, and the number of protein-bound water molecules and ions are estimated by using the block averaging method by Flyvbjerg and Petersen.^84^ The number of blocks is changed until the standard error of the different blocks converges to a constant value. In case the standard error does not converge, the maximum standard error is used as an error estimate. For the dynamic pulling, the error is estimated by calculating the standard error of Δ*F* for each pulling rate.

## Supporting Information Available

Derivation of the standard free-energy of binding from a polymer desorption free-energy profile; additional figures (PDF)
