# Supplementary material for: Quantitative Prediction of Protein–Polyelectrolyte Binding Thermodynamics: Adsorption of Heparin-Analog Polysulfates to the SARS-CoV-2 Spike Protein RBD
Source: JACS Au. 2025 Jan 6;5(1):204–16. doi: 10.1021/jacsau.4c00886 (PMC11775700; doi:10.1021/jacsau.4c00886)
Supplement: Supplementary file 1 — au4c00886_si_001.pdf [file au4c00886_si_001.pdf]

Supporting Information: Quantitative Prediction of Protein–Polyelectrolyte Binding Thermodynamics: Adsorption of Heparin-Analog Polysulfates to the SARS-CoV-2 Spike Protein RBD

Lenard Neander,[†,‡] Cedric Hannemann,[†] Roland R. Netz,[*,†] and Anil Kumar Sahoo[*,†]

†*Department of Physics, Freie Universität Berlin, Arnimallee 14, 14195 Berlin, Germany*

‡*Institute of Chemistry and Biochemistry, Freie Universität Berlin, Takustraße 3, 14195 Berlin, Germany*

E-mail: rnetz@physik.fu-berlin.de; aksahoo@zedat.fu-berlin.de

SI Text

S1. Standard Free-Energy of Binding from a Polymer Desorption Free-Energy Profile

The standard binding free-energy $\Delta F_{\mathrm{b}}^{0}$ of a polymer to a protein as a function of the degree of polymerization N can be obtained from simulations of a shorter polymeric unit N_{sim} as

$$\Delta F_{\mathrm{b}}^{0}(N) = \Delta F_{\mathrm{b}}(N_{\mathrm{sim}}) + \Delta F_{\mathrm{V}} - \Delta F_{\mathrm{stretch}}(N_{\mathrm{sim}}) - T\Delta S_{\mathrm{avidity}}(N, N_{\mathrm{sim}}). \tag{S1}$$

The avidity entropy contribution $\Delta S_{\mathrm{avidity}}(N, N_{\mathrm{sim}}) \approx k_{\mathrm{B}} \ln(N/N_{\mathrm{sim}})$ is only present for $N > N_{\mathrm{sim}} > n_{\mathrm{b}}$, with n_{b} being the number of binding sites on the protein, as explained in the main text. The polymer stretching free-energy $\Delta F_{\mathrm{stretch}}$ is subtracted, as the stretching effect arising from our simulation protocol (see Figure S1) is absent in experimental measurements of equilibrium binding. $\Delta F_{\mathrm{V}}(N_{\mathrm{sim}}) = -k_{\mathrm{B}}T \ln(V_{\mathrm{u}}/V_0)$ represents the free-energy change for transforming from the standard-state volume V_0 ($= 1.661\mathrm{nm}^3$), corresponding to a 1 M concentration, to the sampled volume V_{u} of the unbound state in simulations. The free-energy of binding ΔF_{b} can be obtained from the polymer–protein interaction free-energy profile $F(\mathbf{r})$ as a function of the intermolecular separation vector $\mathbf{r}$ in three dimensions as

$$\Delta F_{\mathrm{b}} = F_{\mathrm{b}} - F_{\mathrm{u}} = -k_{\mathrm{B}}T \ln\left(\frac{Z_{\mathrm{b}}}{Z_{\mathrm{u}}}\right) = -k_{\mathrm{B}}T \ln\left(\frac{\int_{\mathrm{b}} d^3\mathbf{r}\ e^{-F(\mathbf{r})/k_{\mathrm{B}}T}}{\int_{\mathrm{u}} d^3\mathbf{r}\ e^{-F(\mathbf{r})/k_{\mathrm{B}}T}}\right), \tag{S2}$$

where k_{B} is the Boltzmann constant, T is the temperature, and Z_{b} and Z_{u} are the partition functions of the bound and unbound regions, respectively. Since the free-energy profile $F(\xi)$ shown in Figure 2e in the main text is obtained along the reaction coordinate ξ that is aligned to the z-axis, Eq. S2 simplifies to

$$\Delta F_{\mathrm{b}} = -k_{\mathrm{B}}T \ln\left(\frac{\int_{\xi_b} d\xi\ A_b(\xi) e^{-F(\xi)/k_{\mathrm{B}}T}}{\int_{\xi_u} d\xi\ A_u(\xi) e^{-F(\xi)/k_{\mathrm{B}}T}}\right), \tag{S3}$$

where $A_b(\xi)$ and $A_u(\xi)$ are the area available in the bound and unbound region along the orthogonal directions (i.e., x and y) to the reaction coordinate ξ. As $F(\xi)$ is flat in the desorbed state (see Figure 2e in the main text), subtracting the polymer desorption free energy $\Delta F = F(\xi \to \infty)$ from $F(\xi)$ to set $F(\xi) - \Delta F = 0$ in the unbound region implies

$$\Delta F_{\mathrm{b}} = -k_{\mathrm{B}}T \ln \left(\frac{\int_0^{\xi_{\max}} d\xi\ A_b(\xi) e^{-(F(\xi)-\Delta F)/k_{\mathrm{B}}T}}{V_{\mathrm{u}}} \right), \tag{S4}$$

where $V_{\mathrm{u}} = \int_{\xi_u} d\xi\ A_u(\xi)$ is the volume of the unbound state, and $F(\xi) - \Delta F < 0$. Approximating $A_b(\xi)$ with the area of the protein projected on the xy plane, $A_b(\xi) = A_{\mathrm{pro}}$, and defining the ligand binding length as

$$L_{\mathrm{b}} = \int_0^{\xi_{\max}} d\xi\ e^{-F(\xi)/k_{\mathrm{B}}T}, \tag{S5}$$

we obtain from Eq. S4

$$\Delta F_{\mathrm{b}} = -\Delta F - k_{\mathrm{B}}T \ln \left(\frac{A_{\mathrm{pro}} L_{\mathrm{b}}}{V_{\mathrm{u}}} \right) = -\Delta F - k_{\mathrm{B}}T \ln \left(\frac{V_{\mathrm{b}}}{V_{\mathrm{u}}} \right), \tag{S6}$$

where $V_b = A_{\mathrm{pro}} L_{\mathrm{b}}$ is the ligand binding volume. Note that the value of L_{b} is rather insensitive to the cutoff value $\xi_{\max}$ taken to define the bound region, as $F(\xi)$ values for small ξ predominantly contribute to the integral in Eq. S5, see Figure S2. A_{pro} is obtained numerically as described in Figure S3. Inserting the expressions for ΔF_{b}^0 from Eq. S6, ΔF_{V}, and $\Delta S_{\mathrm{avidity}}$ in Eq. S1, we obtain

$$\Delta F_{\mathrm{b}}^0(N) = -\Delta F(N_{\mathrm{sim}}) - k_{\mathrm{B}}T \ln \left(\frac{V_{\mathrm{b}}(N_{\mathrm{sim}})}{V_0} \right) - \Delta F_{\mathrm{stretch}}(N_{\mathrm{sim}}) - k_{\mathrm{B}}T \ln \left(\frac{N}{N_{\mathrm{sim}}} \right), \tag{S7}$$

where the sampled volume V_{u} of the unbound region cancels out. The above final expression is reproduced in the main text as Eq. 5.

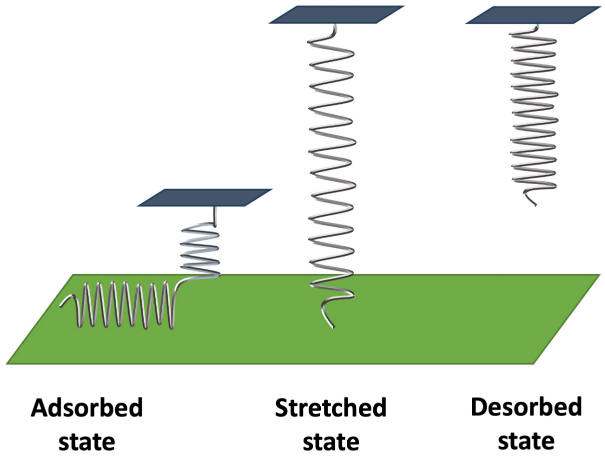

Figure S1: The static pulling simulation protocol leads to the additional stretching free-energy term $\Delta F_{\mathrm{stretch}}$ in Eq. S1.

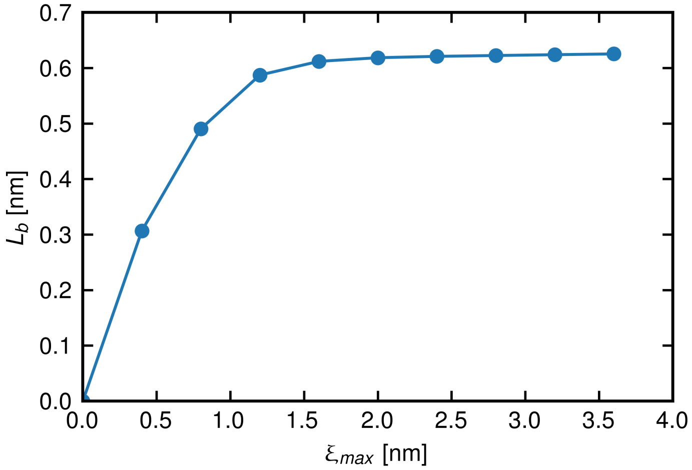

Figure S2: The ligand binding length L_{b} in Eq. S5 as a function of the maximum pulled distance ξ_{max} taken to define the bound region.

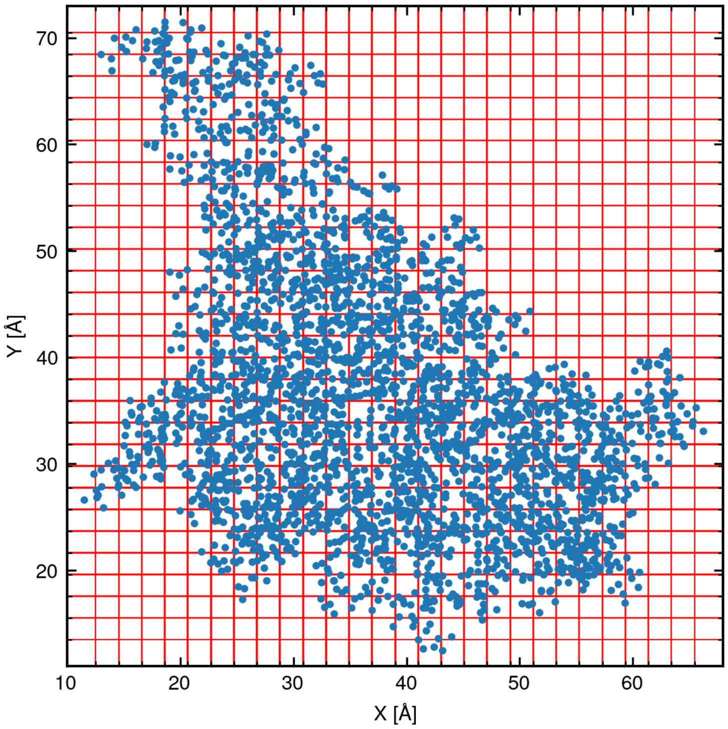

Figure S3: Calculation of the area A_{pro} of the protein projected on the xy plane. The simulation box in the xy plane is divided into 850 square pixels with pixel width 2 Å and the area elements of the pixels containing at least one protein atom (blue dots) are summed to yield the total area $A_{\mathrm{pro}} = 18.1\ \mathrm{nm}^2$.

S2. Comparison of Dynamic-Pulling, Static-Pulling, and Umbrella-Sampling Methods

Both static-pulling and umbrella-sampling methods yield accurate free-energy profiles. The static-pulling method is based on the integration of a force profile, thus obtaining a converged result requires performing simulations at finely spaced windows along a reaction coordinate for time intervals sufficiently greater than the corresponding relaxation times. The umbrella-sampling method, however, is based on probability distributions from a biased sampling of the reaction coordinate by external harmonic potentials, for which there should be sufficient overlap between the probability distributions of consecutive windows to obtain a converged free-energy profile. Therefore, static pulling requires performing long simulations at fewer points along the reaction coordinate, whereas umbrella sampling requires a greater number of finely-spaced windows, for which a shorter simulation time is typically assumed sufficient.

Dynamic pulling can give an equally accurate prediction of the binding free energy but requires sufficiently slow pulling speeds. However, the dynamic pulling method yields friction and diffusion coefficients in the bound protein–polymer complex, which is important for the understanding of the kinetics of the binding process. This information is not available from static pulling or umbrella sampling simulations.

Additional Figures

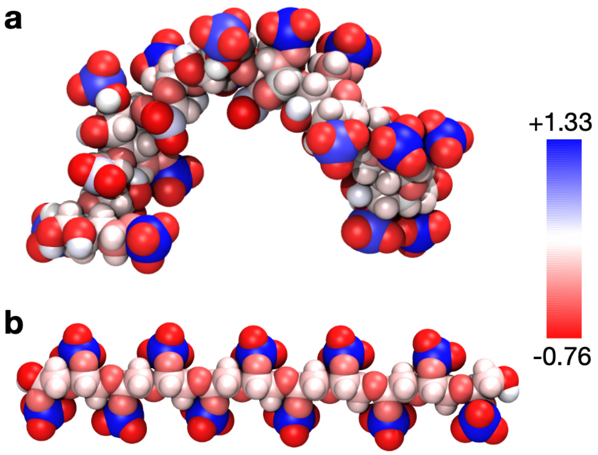

Figure S4: Charge density map for **(a)** the heparin pentamer and **(b)** the LPGS undecamer. A color scale is provided to the right. Values of the linear charge density, $\rho = Q/L_0$, for heparin and LPGS are $\rho_{\mathrm{heparin}} = 4.55$ e/nm and $\rho_{\mathrm{LPGS}} = 3.06$ e/nm.

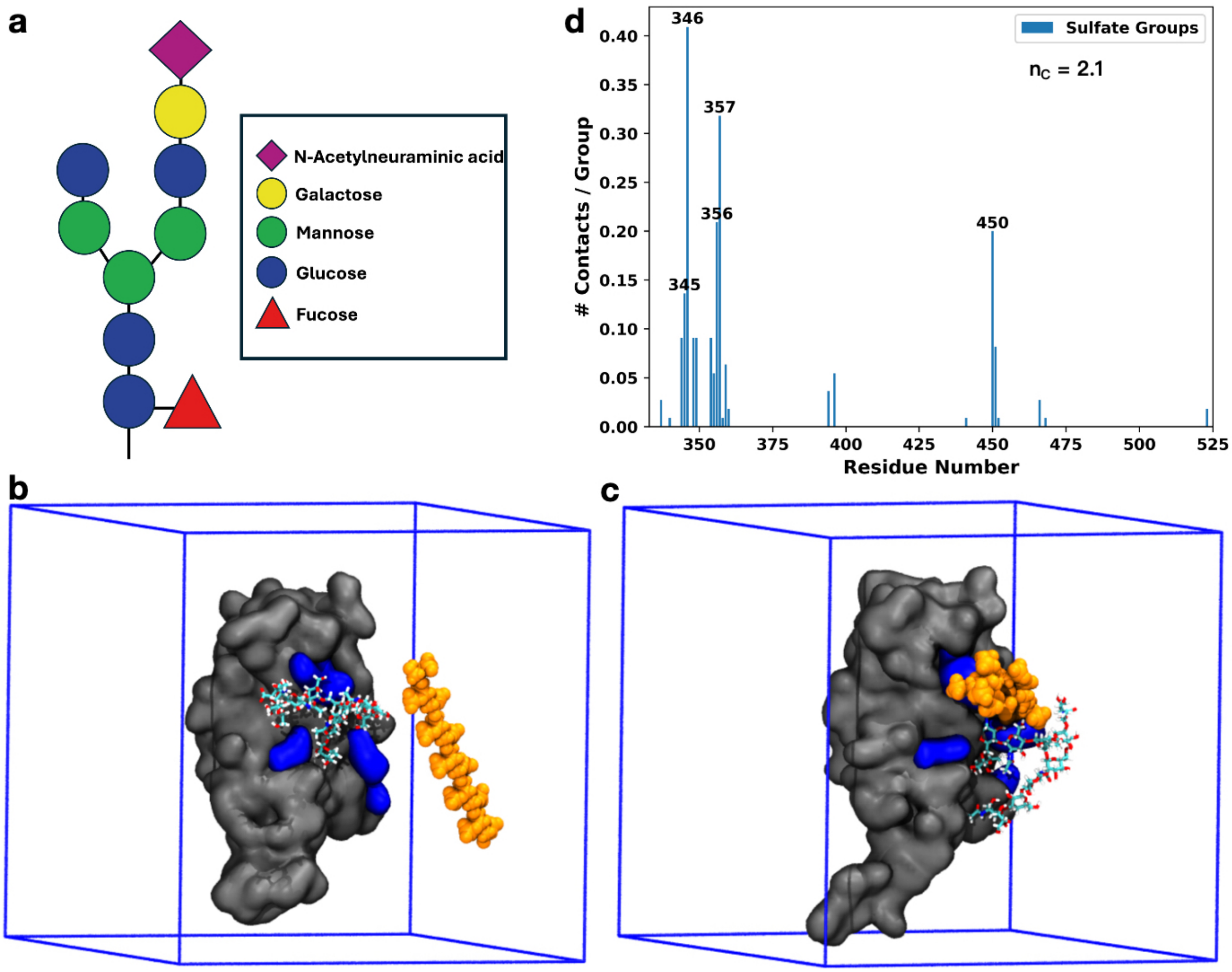

Figure S5: LPGS interactions with the glycan-conjugated RBD. **(a)** Chemical components of the glycan. **(b)** The initial unbound and **(c)** bound configuration of the system after 1.7 μs. LPGS is shown in the space-filling representation in orange, whereas the RBD protein surface is grey. The glycan, shown in the ball–stick representation colored according to atom types, is conjugated to the RBD residue N354 present on top of the cationic patch (highlighted in deep blue). **(d)** Number of close contacts per sulfate group of LPGS with the different amino acid residues of the RBD averaged over the 1.7 μs-long simulation. The total number of such contacts per sulfate group, n_c, is provided in the legend. For a comparison to the simulation without glycan, see Figure S15d.

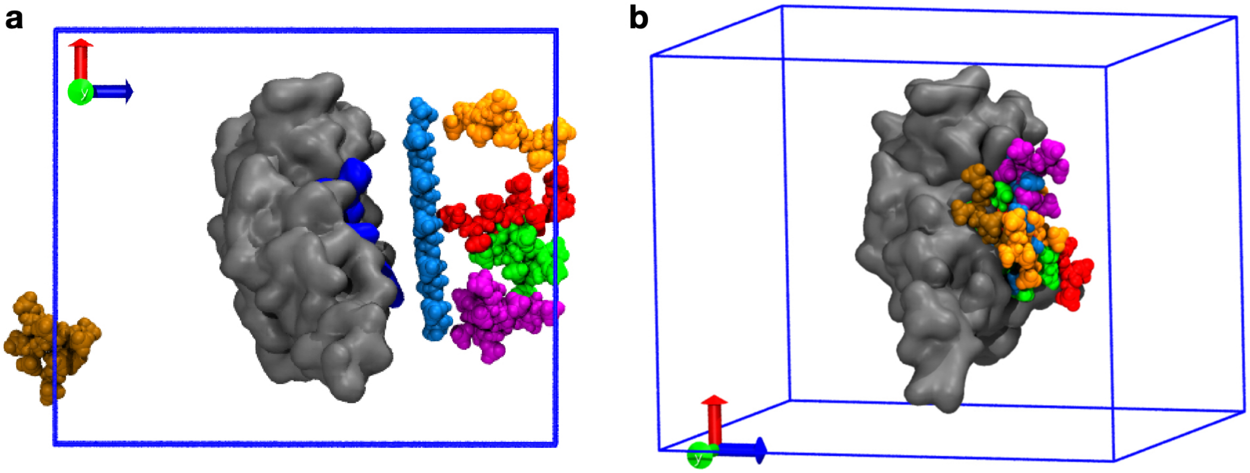

Figure S6: Superimposed **(a)** initial and **(b)** final configurations of LPGS, shown in the space-filling representation in six different colors for the six independent simulations, w.r.t. the RBD protein surface shown in grey. The cationic patch on the RBD with which LPGS primarily interacts in all six simulations is highlighted in deep blue.

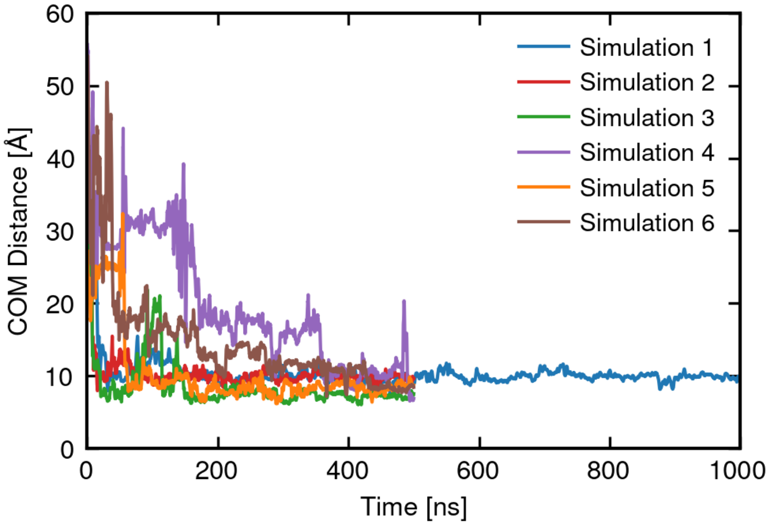

Figure S7: Time series of the distance between the center-of-mass of LPGS and the cationic patch (residues 346, 355, 356, 357, and 466) of the wild-type RBD shown for six independent simulations with different starting positions of LPGS w.r.t. the RBD shown in Figure S6.

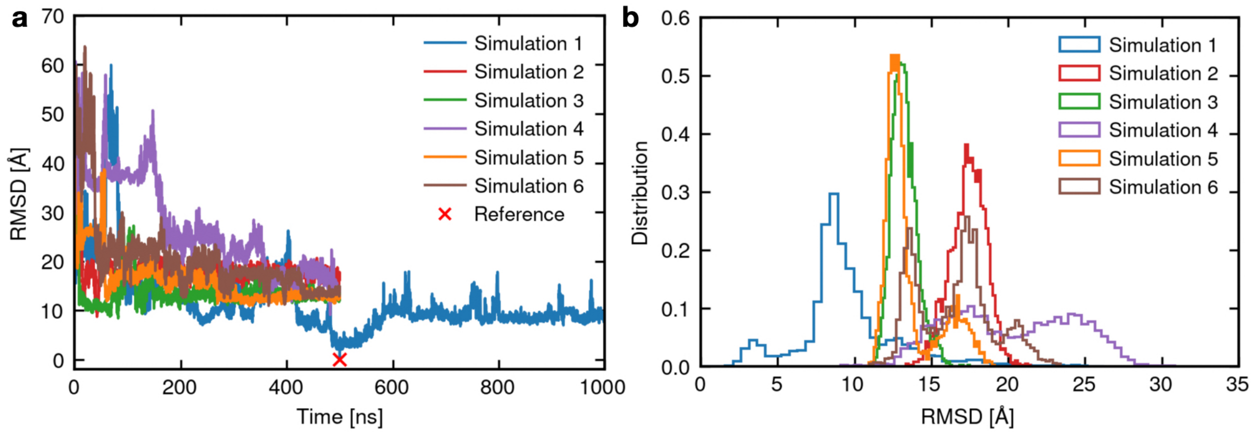

Figure S8: **(a)** Time series of the root-mean-square deviation (RMSD) of configurations of LPGS, w.r.t. the configuration of LPGS bound to the RBD after 500 ns of Simulation-1 (denoted as Reference), in the six independent simulation runs. **(b)** The corresponding normalized distributions, obtained neglecting the first 200 ns of the simulations, show significant overlaps.

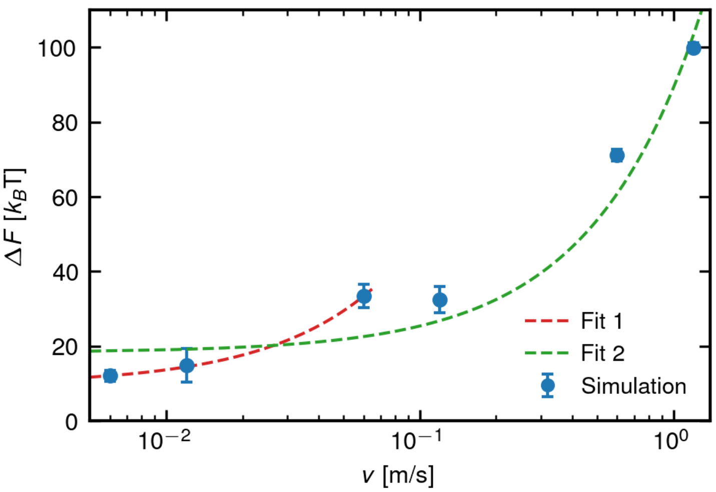

Figure S9: The equilibrium free energy $\Delta F(v = 0)$ of LPGS desorption from the wild-type RBD surface obtained from a linear extrapolation in the pulling velocity v of the dynamic pulling simulation values to $v = 0\,\text{m/s}$. Fit 1 represents the linear extrapolation using data for the lowest three pulling velocities (also shown in Figure 2c in the main text), whereas Fit 2 represents that using all data points. Note the log scale for v. $\Delta F(v = 0)$ from Fit 1 and Fit 2 are $9.7 \pm 1.6\ k_{\mathrm{B}}T$ and $18.3 \pm 1.1\ k_{\mathrm{B}}T$, respectively. The error in $\Delta F(v = 0)$ is from the least square fitting of the data including their error.

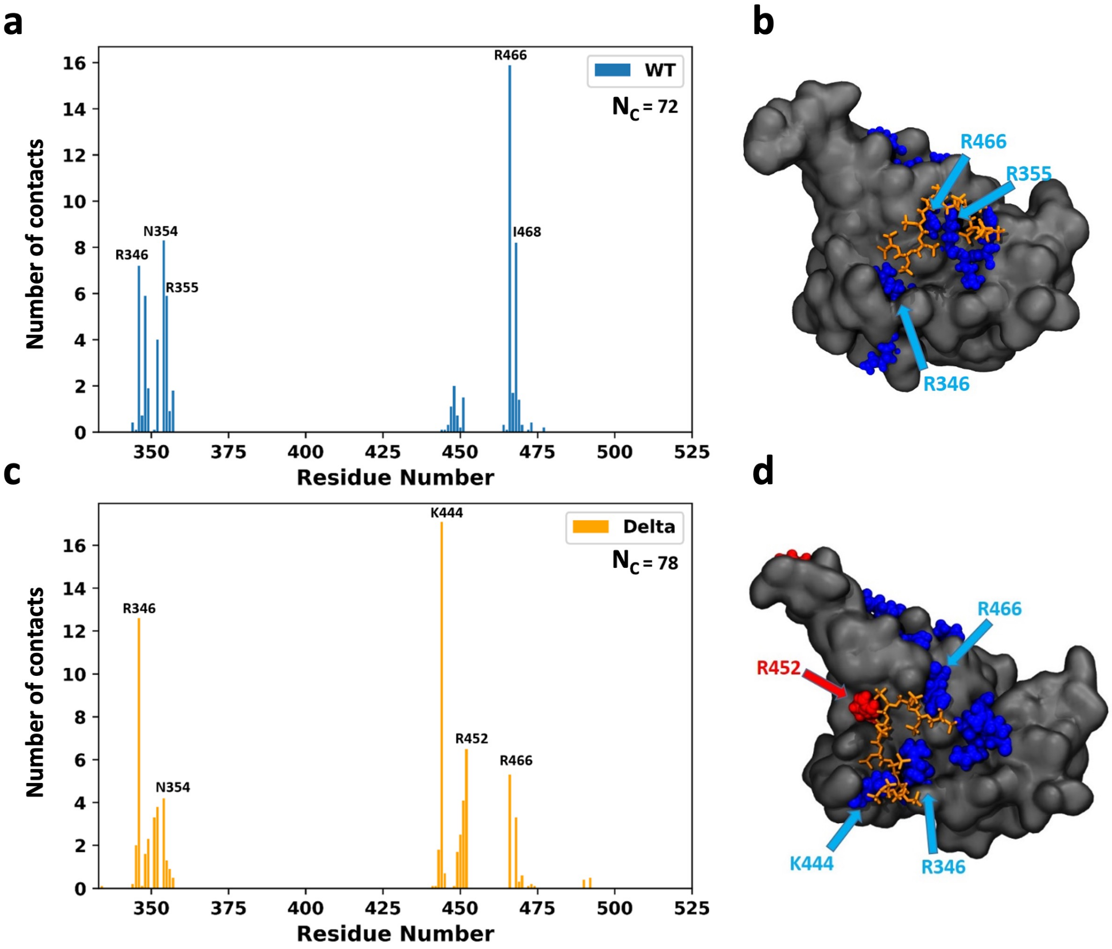

Figure S10: The average number of close contacts of LPGS with different protein residues observed in the 5 µs-long simulation at a pulling distance $\xi = 0.8\,\text{nm}$ of the LPGS terminus from the RBD surface for **(a)** the wild-type and **(c)** the Delta-variant. Representative simulation snapshots for LPGS interactions with **(b)** the wild-type RBD and **(d)** the Delta-variant RBD. LPGS is shown in the ball–stick representation in orange, whereas the protein surface is rendered in grey. Cationic residues of the protein are pointed out in blue and the additional two mutated residues in red, the residues having a substantial number of contacts with LPGS are indicated.

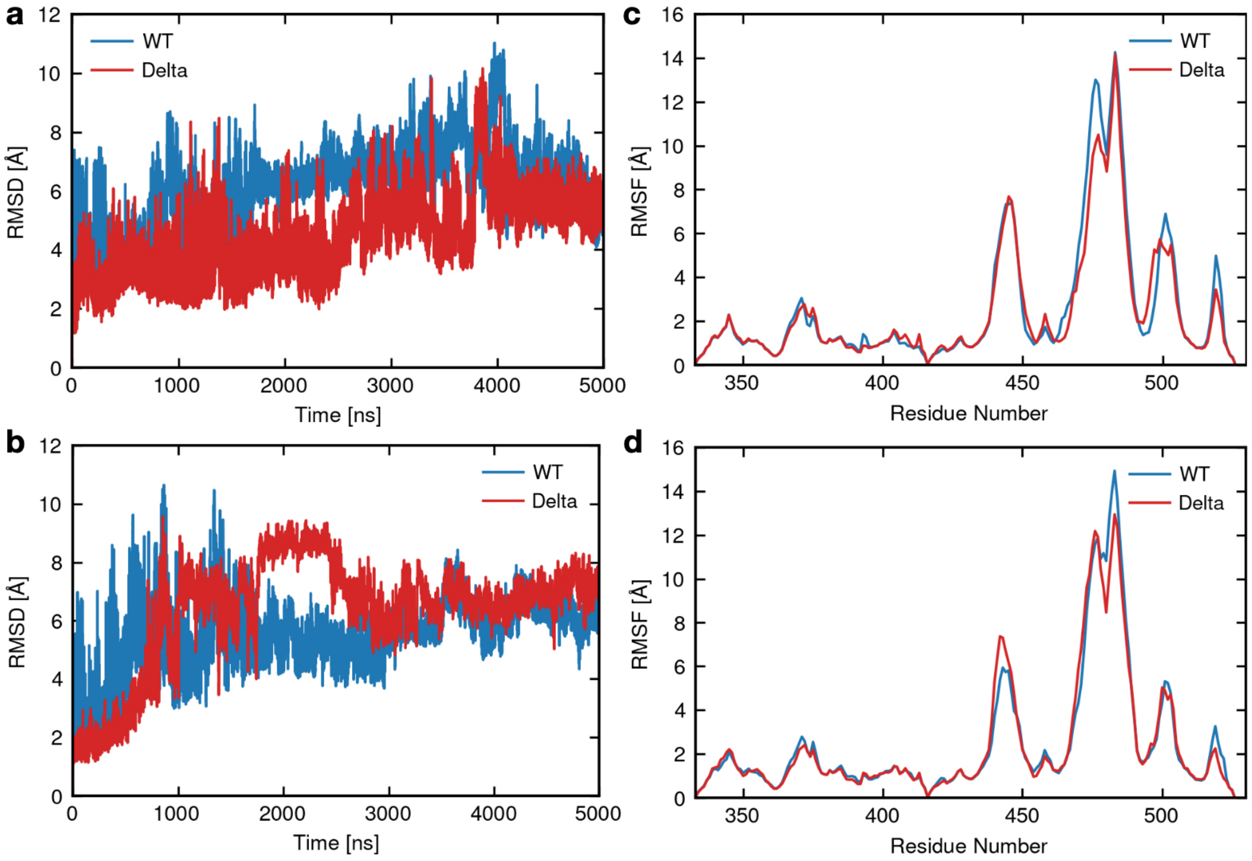

Figure S11: Time-series of the root-mean-square deviation (RMSD) of structures of the wild-type and Delta-variant RBD **(a)** free in solution and **(b)** bound to LPGS w.r.t. the corresponding native structures of the RBD types. Root-mean-square fluctuation (RMSF) of the backbone atoms for different residues of the wild-type and Delta-variant RBD **(c)** free in solution and **(d)** bound to LPGS.

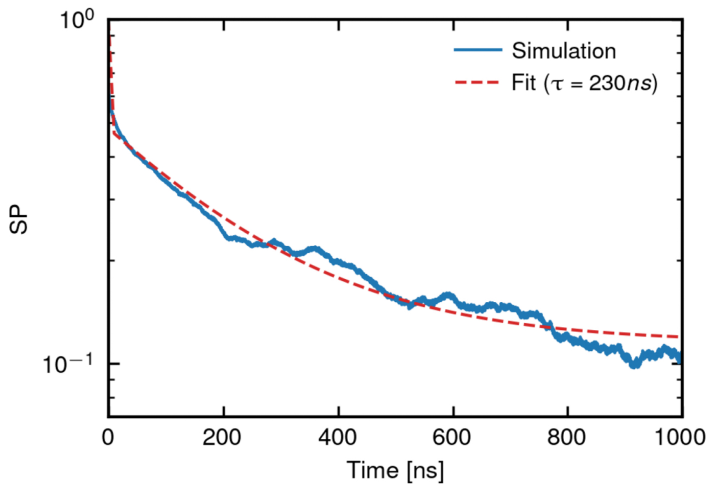

Figure S12: Survival probability for sulfate groups of the LPGS undecamer bound to the Delta-variant RBD surface. The dashed line represents a double exponential fit to the data, with the value of the largest decay time τ given in the legend.

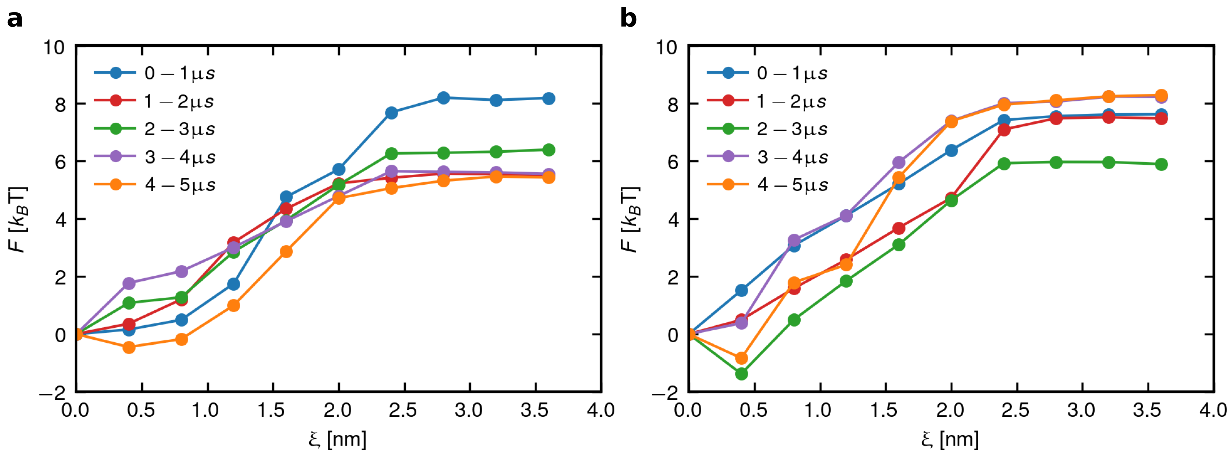

Figure S13: Free energy profiles obtained using data from different blocks (time intervals) of the static pulling simulations for the desorption of LPGS from **(a)** the wild-type RBD and **(b)** the Delta-variant RBD.

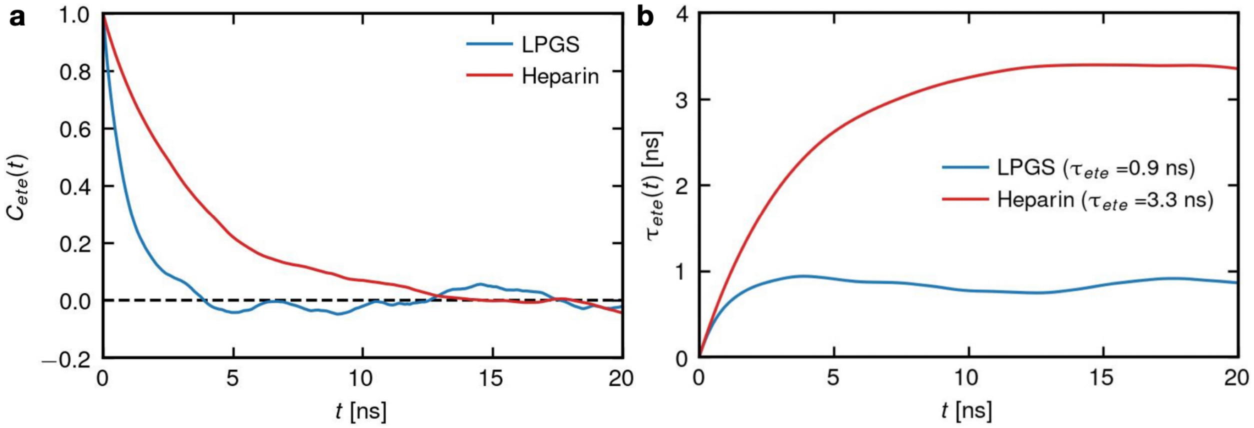

Figure S14: **(a)** End-to-end distance autocorrelation function $C_{ete}(t)$, defined in Eq. 12 in the main text, and **(b)** relaxation time $\tau_{ete} = \lim_{t\to\infty} \tau_{ete}(t) = \lim_{t\to\infty} \int_0^t dt' C_{ete}(t')$, values provided in the legend, for heparin and LPGS.

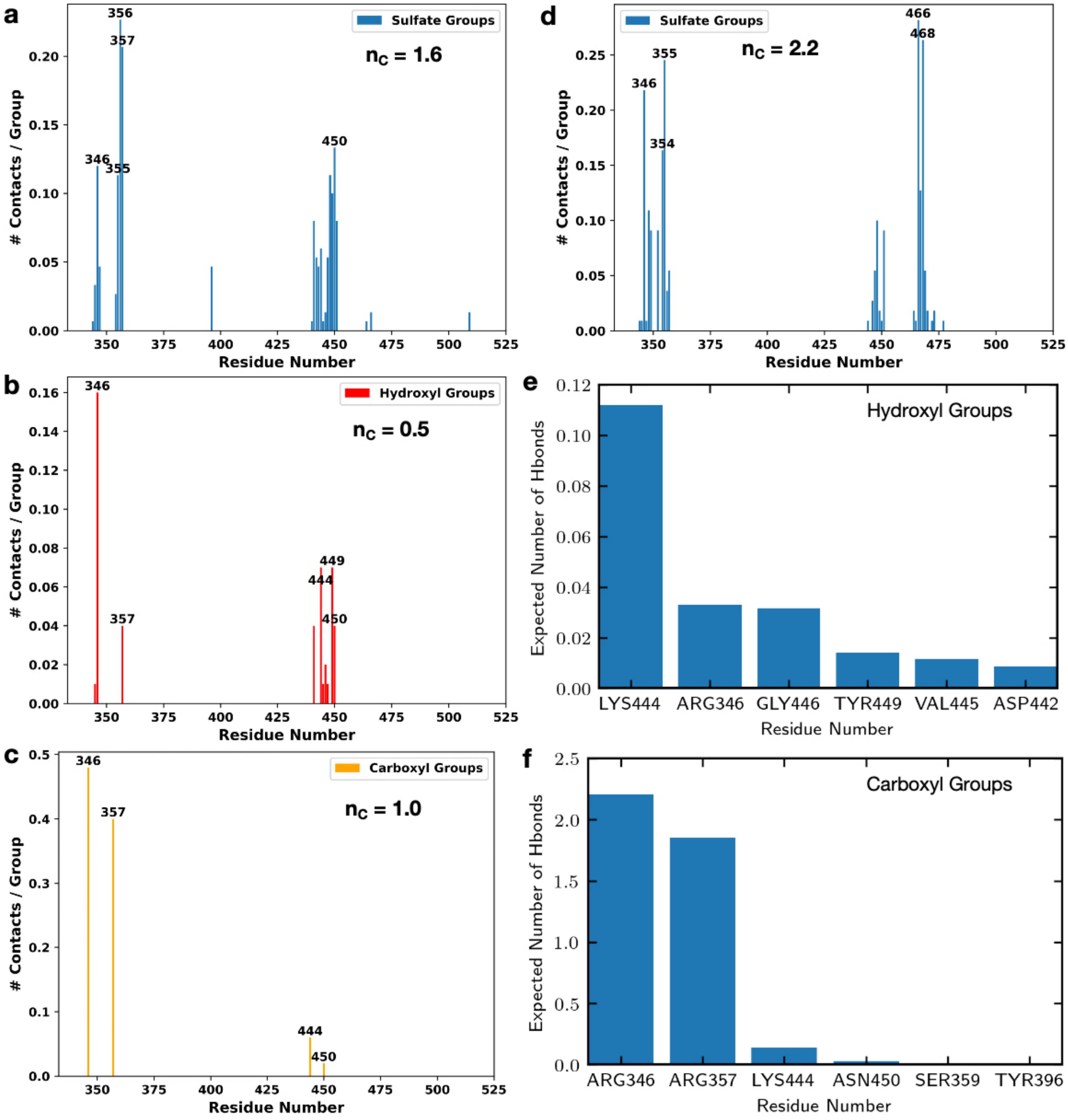

Figure S15: Number of close contacts per **(a)** sulfate, **(b)** hydroxyl, and **(c)** carboxyl group of heparin with the different amino acid residues of the wild-type RBD averaged over 1 μs-long simulation. The total number of such contacts per chemical group, n_c, is provided in the legend. **(d)** The same for sulfates of LPGS. Values for the average number of hydrogen bonds formed by **(e)** hydroxyl and **(f)** of contacts (defined according to the hydrogen bonding criteria) formed by carboxyl groups of heparin with the different amino acid residues of the wild-type RBD are shown in descending order. A hydrogen bond is defined with the criteria of the donor–acceptor distance cutoff 3 Å and the donor–hydrogen–acceptor angle cutoff 150°.

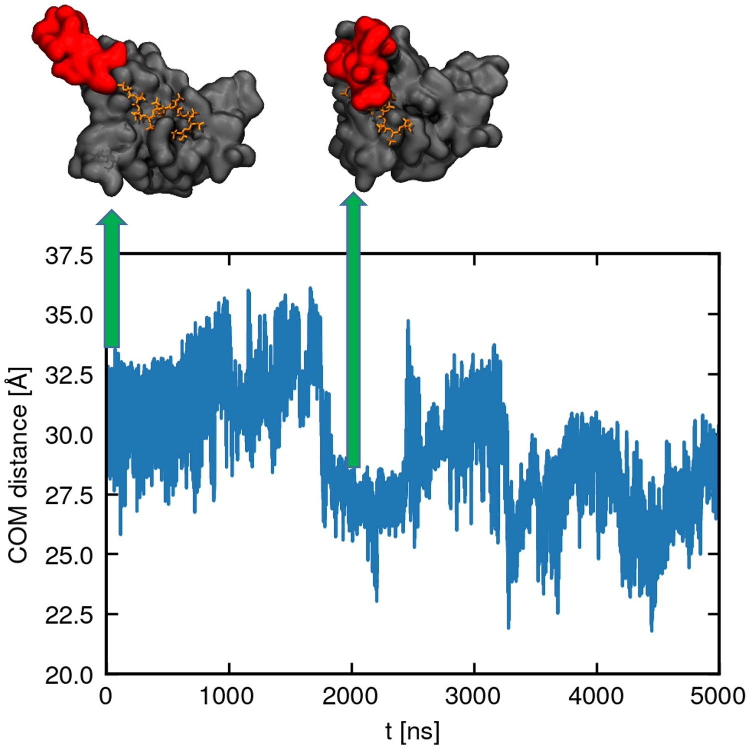

Figure S16: Time series of the distance between the center-of-mass (COM) of the loop region (residues 470 to 490) and the rest of the RBD from the static-pulling simulation of the Delta variant and LPGS at a pulling distance $\xi = 0.4\,\mathrm{nm}$ of the LPGS terminus from the RBD surface. Snapshots of the system at 0 ns and 2000 ns are displayed above. LPGS is shown in orange and the RBD is shown in grey except for the loop region in red. In the time interval of 1800–2800ns, LPGS is found to be entangled between the loop region and the rest part of the RBD, as clear from the snapshot at 2000 ns.

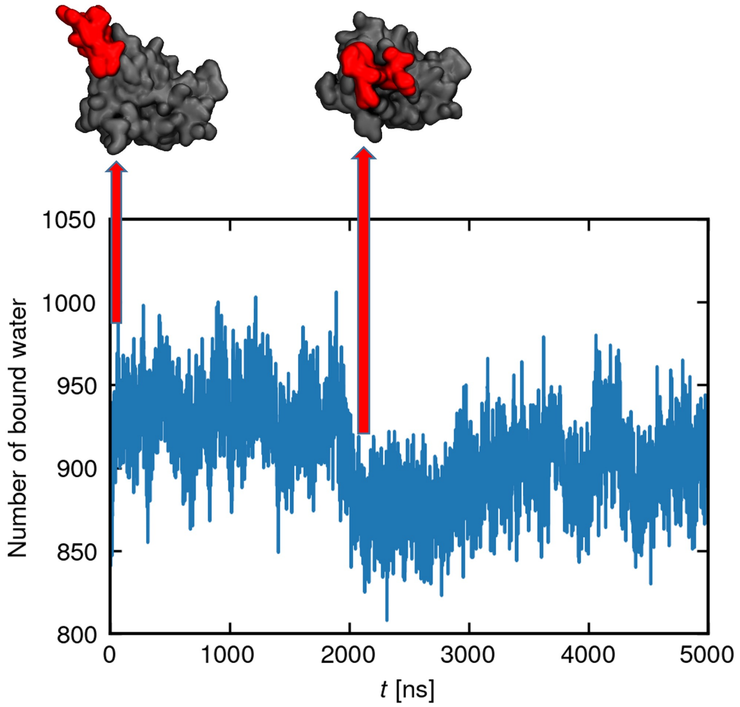

Figure S17: Time series of the number of bound water molecules to protein and LPGS from the simulation of the wild-type RBD at a pulling distance $\xi = 2.8\,\mathrm{nm}$ of the LPGS terminus from the RBD surface. Snapshots at 0 ns and 2000 ns are displayed above the graph. The flexible loop region (residues 470 to 490) of the RBD is shown in red, whereas the rest part is in grey. LPGS is not shown for clarity.

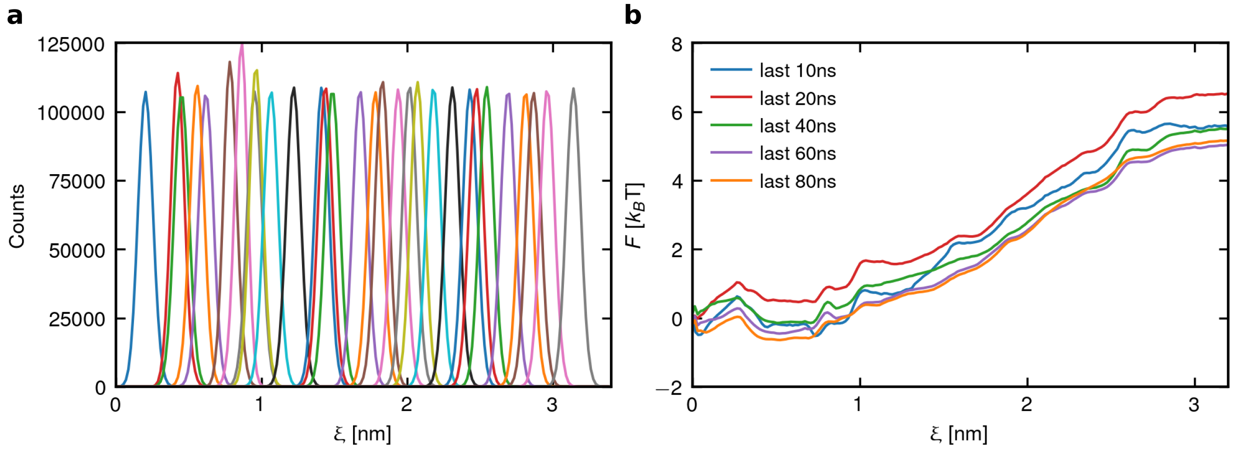

Figure S18: Convergence check for the umbrella sampling simulations for LPGS interactions with the wild-type RBD. **(a)** Histograms of different sampling windows showing nice overlap between consecutive windows. **(b)** Free energy profiles obtained using data from different time intervals of the simulations. The profiles are converged beyond 60 ns of simulation at each window.

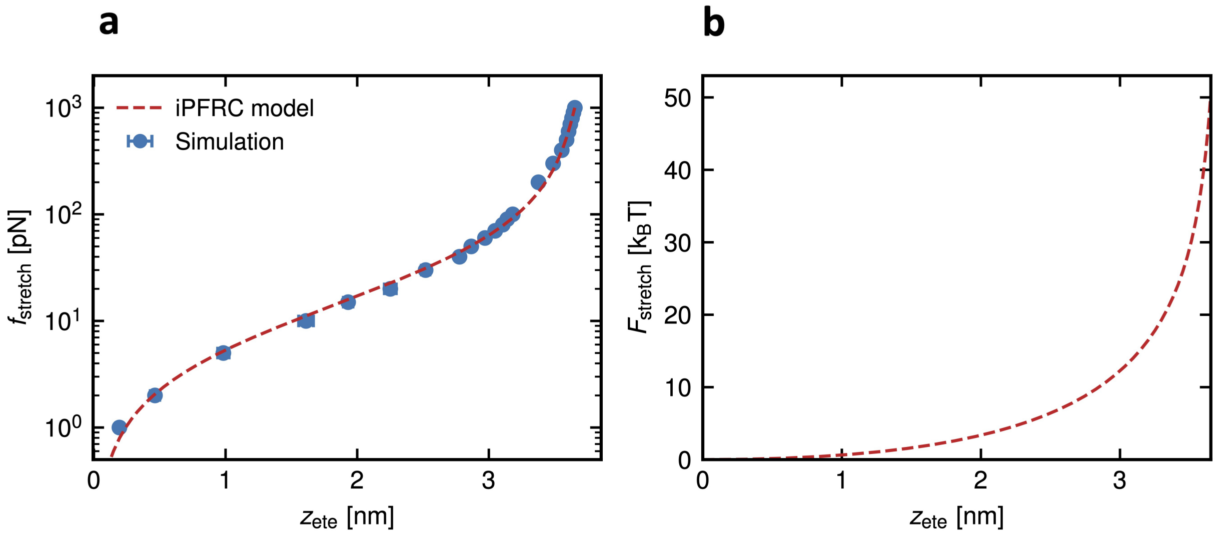

Figure S19: Stretching force and free-energy profiles for an LPGS undecamer. **(a)** Force–extension profile from the stretching simulations fitted with the iPFRC model. Error bars are smaller than the symbol size. **(b)** Stretching free energy obtained by integrating the fitted iPFRC force–extension relation.
